# Roles of conserved active site residues in the IscS cysteine desulfurase reaction

**DOI:** 10.3389/fmicb.2023.1084205

**Published:** 2023-02-16

**Authors:** Yilin Pang, Jing Wang, Xueping Gao, Mengyao Jiang, Lifei Zhu, Feng Liang, Mengxiang Liang, Xiaolin Wu, Xianxian Xu, Xiaojun Ren, Ting Xie, Wu Wang, Qianqian Sun, Xiaojun Xiong, Jianxin Lyu, Jianghui Li, Guoqiang Tan

**Affiliations:** ^1^Zhejiang Provincial Key Laboratory of Medical Genetics, Key Laboratory of Laboratory Medicine, Ministry of Education, School of Laboratory Medicine and Life Sciences, Wenzhou Medical University, Wenzhou, Zhejiang, China; ^2^Hunan Animal Pharmaceutical Company, Hunan Agricultural Group Company, Hunan Agricultural Development & Investment Group Company, Wangcheng Economic and Technological Development Zone, Changsha, Hunan, China; ^3^Department of Laboratory Medicine, The First Affiliated Hospital of Wenzhou Medical University, Wenzhou, China; ^4^People’s Hospital of Hangzhou Medical College, Hangzhou, China

**Keywords:** cysteine desulfurase, pyridoxal 5′-phosphate, active site residues, Cys-aldimine, Cys-ketimine, antimicrobial resistance

## Abstract

*Escherichia coli* cysteine desulfurase (CD), IscS, modifies basal metabolism by transferring sulphur (S) from L-cysteine to numerous cellular pathways, whereas NFS1, a human CD, is active only in the formation of the [Acp]_2_:[ISD11]_2_:[NFS1]_2_ complex. Despite the accumulation of red-coloured IscS in *E. coli* cells as a result of the deficiency of accessible iron, as revealed in our previous studies, the mechanism of the potential enzymatic reaction remains unclear. In this study, the N-terminus of IscS was fused with the C-terminus of NFS1, which was reported to be almost fully active as IscS and exhibits a pyridoxal 5′-phosphate (PLP) absorption peak at 395 nm. Moreover, SUMO-EH-IscS exhibited significant growth recovery and NADH-dehydrogenase I activity in the *iscS* mutant cells. Furthermore, through *in vitro* and *in vivo* experiments combined with high-performance liquid chromatography and ultra-performance liquid chromatography–tandem mass spectrometry, it was shown that the new absorption peaks of the IscS H104Q, IscS Q183E, IscS K206A, and IscS K206A&C328S variants at 340 and 350 nm may correspond to the enzyme reaction intermediates, Cys-ketimine and Cys-aldimine, respectively. However, after mutation of the conserved active-site residues, additional absorption peaks at 420 and 430 nm were associated with PLP migration in the active-site pocket. Additionally, the corresponding absorption peaks of Cys-quinonoid, Ala-ketimine, and Ala-aldimine intermediates in IscS were 510, 325, and 345 nm, respectively, as determined by site-directed mutagenesis and substrate/product-binding analyses during the CD reaction process. Notably, red IscS formed *in vitro* by incubating IscS variants (Q183E and K206A) with excess L-alanine and sulphide under aerobic conditions produced an absorption peak similar to the wild-type IscS, at 510 nm. Interestingly, site-directed mutation of IscS with hydrogen bonds to PLP at Asp180 and Gln183 resulted in a loss of enzymatic activity followed by an absorption peak consistent with NFS1 (420 nm). Furthermore, mutations at Asp180 or Lys206 inhibited the reaction of IscS *in vitro* with L-cysteine (substrate) and L-alanine (product). These results suggest that the conserved active site residues (His104, Asp180, and Gln183) and their hydrogen bond with PLP in the N-terminus of IscS play a key role in determining whether the L-cysteine substrate can enter the active-site pocket and regulate the enzymatic reaction process. Therefore, our findings provide a framework for evaluating the roles of conserved active-site residues, motifs, and domains in CDs.

## 1. Introduction

*Escherichia coli* IscS is a PLP-dependent homodimeric enzyme that catalyses the conversion of L-cysteine into L-alanine and sulphur (Yang et al., 2015). Based on the crystal structure of IscS, the PLP cofactor was identified inside the active site pocket near the surface of the protein. The pocket is made up of several charged or polar amino acid side chains, including His104, Lys105, Asn155, Glu156, Tyr337, and Arg354 (Cupp-Vickery et al., 2003). The imidazole ring of His104 is situated above the pyridoxal ring in this active site and is hypothesized to act as an acid–base catalyst in several protonation and deprotonation processes during catalysis (Kaiser et al., 2000). Additionally, the phenolate oxygen and pyridine N1 of PLP can generate hydrogen bond interactions with Gln183 and Asp180 of IscS, respectively, during the PLP pocket-binding process (Cupp-Vickery et al., 2003). Hydrogen bond interactions that occur between Asp79 and Asp180 likely impair the electron-withdrawing interaction of Asp180 with the pyridine N1 of PLP, thereby affecting the stability and conversion rates of intermediates during the PLP reaction cycle (Cupp-Vickery et al., 2003). IscS comprises a conserved lysine (Lys206) and a catalytic cysteine residue (Cys328), both of which are indispensable for the catalytic activity of the enzyme (Shi et al., 2010). The formation of an internal aldimine Schiff base with Lys206, as well as several polar and nonpolar interactions, anchor the PLP cofactor to the active site. Cys328 is a catalytic residue that contributes to the formation of the Cys328-persulphide group (-SSH) *via* nucleophilic attack of the PLP-bound L-cysteine substrate (Cupp-Vickery et al., 2003; Ikeuchi et al., 2006).

Cysteine desulfurases (CDs) are classified into two types based on primary amino acid sequence comparisons: type I (NifS and IscS) and type II (SufS; Blahut et al., 2019). In the resting state, Nakamura et al. (2020) successfully captured *Helicobacter pylori* NifS and the PLP-L-cysteine intermediate. The α-carboxy group of the L-cysteine substrate was noncovalently bound to the guanidium moiety of the conserved Arg354 residue *via* polar interactions in the PLP-L-cysteine intermediate structures. Furthermore, by orienting the thiol moiety of PLP-L-cysteine towards the solvent area, the polar interaction between the conserved His104 residue and the thiol group of PLP-L-cysteine could facilitate nucleophilic attack by cysteine residues (Cys328) on the catalytic loop. These reports suggest that in addition to the conserved Lys206 and Cys328 sites, other conserved active-site residues are also important in the enzymatic reaction of CDs.

Persulfide becomes sufficiently mobilised by interacting with other proteins to be incorporated either directly or indirectly into various thiocofactors (such as, iron–sulphur clusters, biotin, lipoic acid, thiamine, and molybdopterin; Lauhon and Kambampati, 2000; Zhang et al., 2010). Thio-cofactor-binding proteins have important and diverse roles in cellular processes such as epigenetic regulation, gene expression, respiration, intermediary metabolism, and redox sensing (Tan et al., 2014; Das et al., 2021). CDs are also involved in maintaining iron homeostasis in organisms as well as tRNA thiolation and DNA phosphorothioate modification (Shi et al., 2010; An et al., 2012). More importantly, previous studies have demonstrated that CD modulates the pathogenesis, antimicrobial resistance (AMR), and survival of several pathogenic microbes within their hosts (Das et al., 2021).

**Figure 1:**
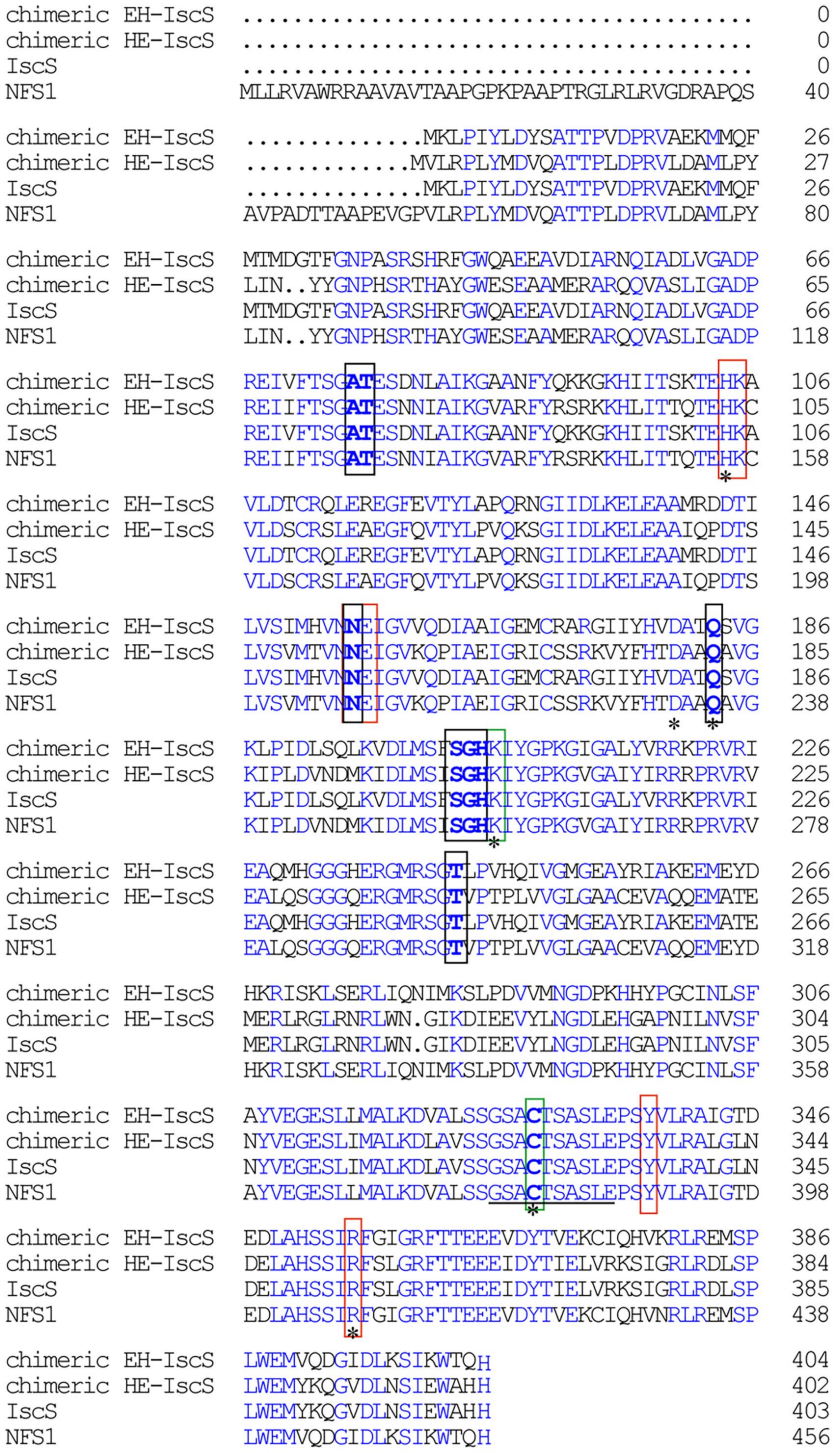
Sequence alignment of *Escherichia coli* IscS, human NFS1, chimeric CD EH-IscS, which was constructed by fusing the N-terminal domain of *E. coli* IscS with the C-terminal domain of human NFS1, and chimeric CD HE-IscS, which was constructed by fusing the N-terminal domain of NFS1 (55–457) with the C-terminal domain of IscS. Amino acid sequence alignment was performed with *DNAMAN*. Blue areas indicate identity, black frames indicate the conserved pyridoxal 5′-phosphate (PLP) binding sites, and red frames represent the pocket. A line (325–334) indicates the mobile active site loop of IscS that is proposed to be involved in the sulphur transfer. The green frames represent two highly conservative amino acid residues of IscS. Mutated amino acid residues (His104, Asp180, Gln183, Lys206, Cys328, and Arg354) are marked with an asterisk.

The CD is highly conserved in both prokaryotic and eukaryotic organisms (Figure 1; Bühning et al., 2017). In contrast to IscS, human NFS1 requires the adaptor protein ISD11 and the acyl carrier protein (Acp) to maintain its activity (Cai et al., 2017). NFS1 and ISD11 are mitochondrial proteins (Friemel et al., 2017), and mutations in these proteins can lead to rare but severe mitochondrial disorders (Farhan et al., 2014; Saha et al., 2015). Additionally, functionally producing these proteins in *E. coli* is particularly challenging because majority of the over-expressed protein is sequestered in inclusion bodies (Marelja et al., 2008; Li et al., 2018).

In recent years, CDs have been identified and refined in the catalytic process of intermediate formation (Behshad and Bollinger, 2009; Yang et al., 2015; Blahut et al., 2019; Nakamura et al., 2020), based on which, a possible chemical mechanism for IscS was proposed: the first intermediate is a gem-diamine complex formed by the nucleophilic attack of the amine group of L-cysteine on the PLP cofactor, and these species can accumulate (Phillips et al., 1990). The second intermediate is a stable complex attributeable to the Cys-aldimine and Cys-ketimine forms in rapid equilibrium. Among them, Cys-aldimine is converted to a short-lived Cys-quinonoid intermediate, followed by Cys-ketimine. In rapid equilibrium, the third intermediate comprises the Ala-ketimine, Ala-quinonoid, and Ala-aldimine forms. This is due to the sulfhydryl group (-SH) of the enzyme activity site Cys-328, which is activated to form sulphur anions. It then targets the sulfhydryl of Cys-ketimine *via* a nucleophilic attack, resulting in the cleavage of the C-S bond and persulfide formation (Cys328-S-SH). Persulfide is a sulphur donor involved in the biosynthesis of iron–sulphur clusters and other cofactors. Subsequently, the alanine product is released and the internal aldimine regenerated between PLP and Lys-206. Importantly, when the PLP cofactor is occupied by the second L-cysteine, it is anticipated to inhibit persulfide cleavage, thereby promoting substrate inhibition in the steady state (Behshad and Bollinger, 2009). Several important acid–base catalyses (deprotonation and protonation) steps are involved in the desulfurase reaction. However, the exact active site residues that carry out these acid–base catalysis steps remain unclear.

Our previous study showed that IscS purified from wild-type *E. coli* MC4100 cells is yellow because it contains a PLP cofactor. However, the IscS expressed in the *E. coli* ∆*iscA*∆*sufA* mutant strain turned red. Furthermore, the depletion of intracellular iron also leads to the accumulation of red IscS in wild-type *E. coli* cells, and the purified red IscS has an absorption peak at 528 nm, in addition to the PLP peak at 395 nm. Moreover, the active catalytic sites of Lys206 and Cys328 are required for the formation of red IscS in the *E. coli* iscA/sufA mutant cells. Notably, purified IscS-K206A has two new absorption peaks at 338 and 428 nm (Yang et al., 2015). Previous studies have also shown that the enzymatic reaction of CDs is a multistep reversible reaction (Behshad and Bollinger, 2009; Blahut et al., 2019). It is, therefore, necessary to explore the role of the other conserved active site residues in the enzymatic reaction of IscS and analyse whether the new absorption peaks in the IscS mutants are the intermediates of the enzymatic reaction, to lay a theoretical foundation for further elaborating the mechanism of red IscS accumulation and its physiological significance.

The aim of this study was to elucidate the chemical mechanism of the formation of the cysteinyl persulfide intermediates in the IscS reaction process, as well as to demonstrate CD activity deficiency in human NFS1. First, we constructed two chimeric CDs derived from IscS and NFS1. Second, the chemical mechanism of the IscS reaction was dynamically dissected using site-directed mutation of its active site residues (H104Q, D180G, Q183E, K206A, C328S, and R354K). To determine the function of conserved active site residues in IscS activity and the accumulation of intermediates in the IscS process, UV–visible absorption, substrate/product binding, and enzymatic activity experiments were performed. Furthermore, the physiological significance of red quinonoid intermediate accumulation was elucidated using CD activity assay and iron–sulphur cluster assembly experiments. In particular, the molecular weights and chemical structural properties of several intermediates were identified and analysed using high-performance liquid chromatography (HPLC), fluorescence spectrometry, and ultra-performance liquid chromatography–tandem mass spectrometry (UPLC-MS). These results are critical for elucidating the role of conserved active site residues in the IscS enzymatic reaction, as well as the steps of the IscS enzymatic reaction.

## 2. Materials and methods

### 2.1. Strains, plasmids, and reagents

pCold I and pCold TF vectors, as well as restriction endonucleases, were purchased from Takara Corporation (Dalian, China). DNA polymerase and T4 DNA ligase were obtained from Vazyme Biotechnology Corporation (Nanjing, China). The ClonFast kit was acquired from Obio Technology Corporation (Shanghai, China). 2,2′-Dipyridyl, DTT, L-cysteine, L-alanine, L-glutathione reduced (GSH), and deamino-NADH were purchased from Merck (Darmstadt, Germany). Pyridoxal-5′-phosphate was obtained from Sangon Biotechnology Corporation (Shanghai, China). All primers were synthesised by BGI (Shenzhen, China). The remaining chemicals were of analytical grade.

### 2.2. Construction of recombinant plasmids and site-directed mutagenesis

The recombinant plasmids were constructed as previously described (Yang et al., 2015; Li et al., 2018). The *iscS* gene was amplified from the *E. coli* MC4100 genome. Each terminal of the amplified *iscS* gene contains homologous arms, which are the same as those of the pCold I and pBAD/HisD plasmids digested with *Kpn* I. The IscS-pCold I and IscS-pBAD/HisD plasmids were constructed using Seamless Cloning Technology (SCT). EH-IscS included the N-terminal domain amino acids 1–263 of IscS and the C-terminal domain of NFS1 (amino acids 316–457), whereas HE-IscS included the N-terminal domain of NFS1 (amino acids 55–315) and the C-terminal domain of IscS (amino acids 264–404). The plasmids pCold I and pCold TF were digested with *Kpn* I, whereas pCold-SUMOa and pBAD/His SUMO expression vectors were digested with *Nde* I, and all recombinant plasmids were constructed using the SCT mentioned above.

In this study, we selected six (active-site) residues associated with PLP binding to perform site-directed mutagenesis, namely His104, Asp180, Gln183, Lys206, Cys328, and Arg354. A 15–20 bp sequence was selected as the homology arm on the mutation site and plasmid backbone, respectively, and the mutated site base was contained in the homologous arm. Moreover, using the IscS-pBAD/Myc-HisC plasmid as a template, two DNA fragments amplified using two pairs of primers were designed based on two homologous arms. In addition, forward and reverse primers based on the homology arms in the plasmid backbone were suitable for the construction of all site-directed mutagenesis plasmids. All the site-directed mutant plasmids were constructed using SCT. A double mutant, IscS K206A&C328S, was obtained through an additional round of site-directed mutagenesis. The sequences of the plasmid constructs were confirmed by DNA sequencing (BGI, Shenzhen, China).

### 2.3. Protein expression

Wild-type *E. coli* IscS and IscS variants, chimeric CDs, and NFS1 were expressed in *E. coli* wild-type MC4100 or *E. coli* BL21(DE3) cells. *E. coli* cells, hosting the pBAD/HisD and pBAD/His SUMO expression plasmids, were grown in Luria-Bertani (LB) medium and incubated with or without 2,2′-dipyridyl (DP). When the cells reached an OD_600_ of 0.6 under aerobic conditions, arabinose (at a final concentration of 0.02%) was added to the cell cultures to induce the expression of recombinant IscS, IscS variants, and chimeric CDs, followed by cell incubation at 37°C for 3–6 h or 25°C overnight-24 h, respectively.

### 2.4. Purification of recombinant CDs by Ni-NTA column

Purification of recombinant CDs was conducted as previously described by Yang et al. (2015). Briefly, *E. coli* cells were induced and harvested after recombinant protein expression; the cell precipitates were re-suspended in a certain volume of protein purification buffer A containing Tris (20 mM, pH 8.0) and NaCl (500 mM). The cell suspension was then passed through a low-temperature ultra-high-pressure continuous-flow cell disrupter (JN-3000 PLUS, China) three times. After centrifugation at 25,000 rpm for 40 min, the supernatant was collected for protein purification. The proteins were purified using the Ni-agarose column (Qiagen Co.), followed by purification using a gel filtration column (SuperdexTM 75 10/300GL, GE). Buffer A was used to balance the Ni-agarose column and elution buffer B was used to elute the target protein. Elution buffer B was composed of buffer A, containing imidazole (500 mM). The purity of all proteins was greater than 95%, as demonstrated by the sodium dodecyl-sulfate polyacrylamide gel electrophoresis (SDS-PAGE) gel stained using Coomassie blue. CD protein concentrations were measured from the absorption peak at 280 nm and calculated using CD extinction coefficients. The extinction coefficient of the CD protein was calculated by inputting its amino acid sequence into a Peptide Property Calculator.^1^ The extinction coefficients of IscS, EH-IscS, SUMO-EH-IscS, TF-EH-IscS, SUMO-NFS1 (55–457), SufS, and SufE were 39.8, 34.3, 35.6, 50.2, 38.4, 48.13, and 21.15 mM^−1^ cm^−1^, respectively. UV–visible absorption spectra were measured using a Hitachi U3900 UV–vis spectrometer equipped with a temperature controller.

### 2.5. Growth curve

The overnight cultures were diluted to OD_600_ = 0.02 with fresh LB medium (50 ml) containing 0.002% arabinose. The cells were grown at 37°C for 10 h and monitored by measuring cell density at 600 nm every 2 h.

### 2.6. Enzyme activity assay for NADH dehydrogenase I

NADH dehydrogenase I activity in *E. coli* cells was measured as previously described (Tan et al., 2014). The overnight cultures were diluted to OD_600_ = 0.02 with LB medium, and cells were grown to OD_600_ = 0.6. The cells were washed with saline solution after centrifugation and resuspended to an OD_600_ of 20. Inverted membrane vesicles of *E. coli* cells were prepared by passing the cells through a low-temperature ultra-high-pressure continuous-flow cell disrupter (JN-3000 PLUS). NADH dehydrogenase I activity was measured using deamino-NADH as the specific substrate. Inverted membrane vesicles (20 μl) were added to a reaction solution containing Tris (20 mM, pH 8.0), NaCl (200 mM), and deamino-NADH (100 μM). NADH dehydrogenase I activity was determined by measuring the oxidation of deamino-NADH at 340 nm and 37°C.

### 2.7. The CD activity assay

CD activity assay was conducted as previously described (Siegel, 1965; Yang et al., 2015; Li et al., 2018). Briefly, purified recombinant CDs (5 μM) were incubated with buffer A in the presence of 3 mM dithiothreitol (DTT) for 5 min at 37°C. L-cysteine (2 mM) was added to initiate the CD activity reaction. Reactions were terminated by the addition of 20 mM N, N-dimethyl-p-phenylene-diamine sulphate (in 7.2 M HCl), and 30 mM FeCl_3_ (in 1.2 M HCl). The colour was left to develop for 20 min at 37°C before quantifying methylene blue at 669 nm. Buffer A was used as the negative control and endonuclease III (Nth) was used as the positive control. The iron content in *E. coli* Nth was calculated from the iron-ferrozine determination, as previously described by Ren et al. (2021), and because *E. coli* Nth contains a stable [4Fe-4S] cluster, the protein should have equal amounts of iron and sulphur. Therefore, the extinction coefficient of sulphur was calculated based on the content of iron in Nth. Spectra were recorded every 5 min for 15 min.

### 2.8. Substrate- or product-binding assay

The substrate- or product-binding assay was conducted as previously described by Li et al. (2018).

### 2.9. UPLC-MS

An ABSciex 6500 plus QTRAP (SCIEX, United States) mass spectrometer coupled to a ExionLC AD UPLC System (SCIEX, United States) was employed for this study. A symmetry C18 column (2.1 mm × 150 mm, 3.5 μM) was used for the chromatographic separation of PLP and its derivatives. The flow rate was 0.3 ml/min, and the injection volume was 2 μl. Mobile phase A was 10 mM NH_4_OAC solution (pH 5.6); mobile phase B was acetonitrile solution. The gradient elution procedure was as follows: 0–1 min, 90% A and 10% B; 1.1 min, 10% A and 90% B; 1.1–5 min, 10% A and 90% B; 5.1 min, 90% A and 10% B; 5.1–6 min, 90% A and 10% B. PLP and its derivatives were measured by electrospray ionization (ESI) in positive ionization mode, the data were collected by full scanning (scanning mass range *m*/*z* was 100–400), and the parent ions of the compound were preliminarily determined. Other mass spectrometer settings were: nitrogen gas pressure as a curtain (CUR), 20 psi; temperature, 350°C; ion spray voltage, 5,500 volts; ion source (GS1), 40 psi; GS2, 40; collision exit potential, 95 V; entrance potential, 10 V. Data were collected/processed using AB Sciex Analyst software version 1.7/Multiquant version 3.02.

### 2.10. Iron-sulfur cluster assembly in *Escherichia coli* IscU

Purified IscU (100 μM) was incubated with Fe(NH_4_)_2_(SO_4_)_2_ (100 μM), yellow IscS (Y; 0.5 μM) or red IscS (R; 0.5 μM), DTT (3 mM), NaCl (200 mM), and Tris (20 mM, pH 8.0) under aerobic conditions. L-cysteine (3 mM) was added after 2 min of preincubation at 37°C. UV–visible absorption spectra were recorded every 2 min for 16 min.

### 2.11. Data analysis

All data are expressed as the mean ± standard deviation (SD) and were analysed using SPSS software (version 16.0). Differences between mean values were evaluated using a one-way analysis of variance and Tukey’s multiple comparison test (if applicable). A *p*-value less than 0.05 was considered statistically significant.

## 3. Results

### 3.1. The N-terminus of the *Escherichia coli* IscS is crucial for its activity, and chimeric EH-IscS can restore cell growth of the △*iscS* single mutant

**Figure 2:**
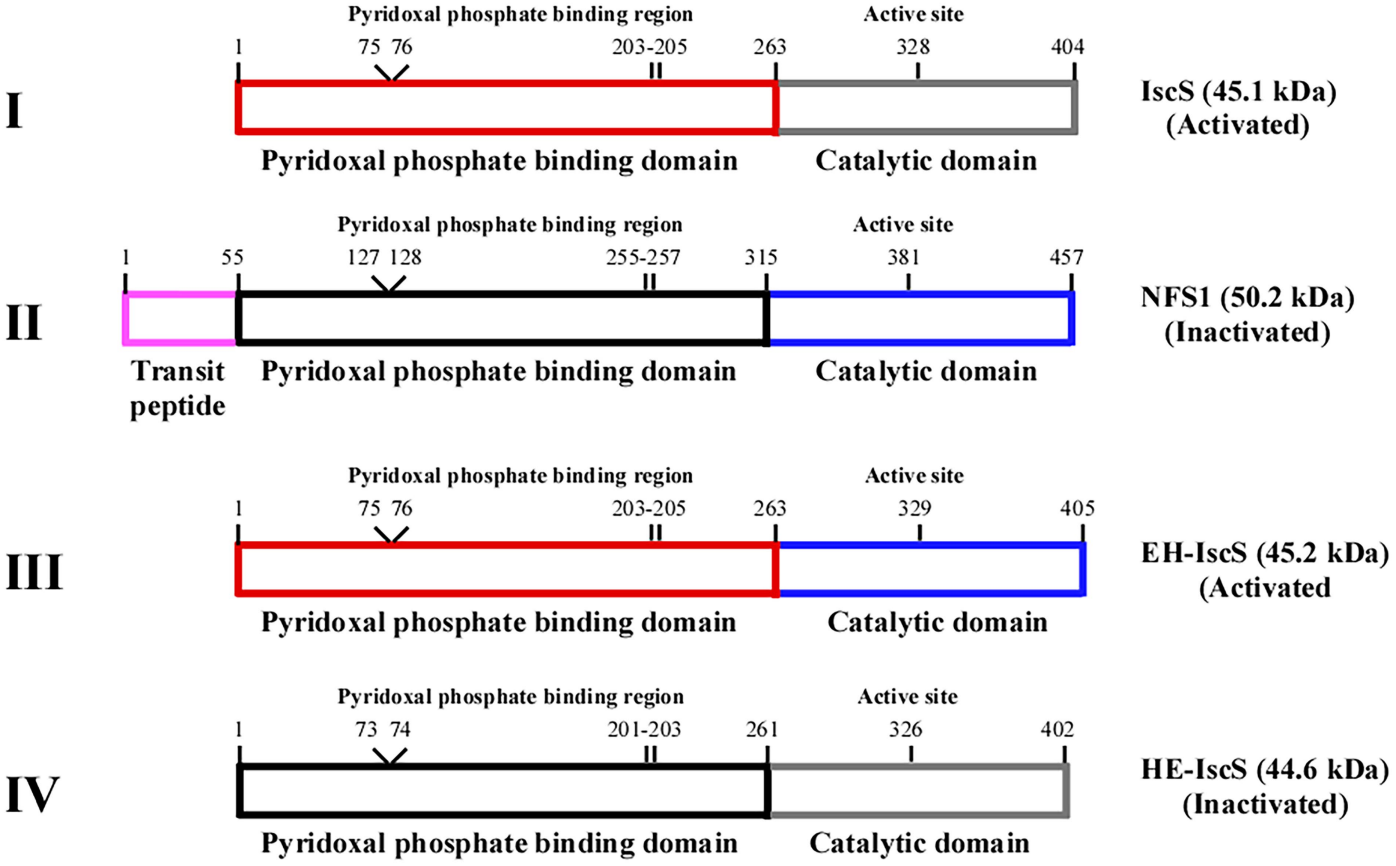
Schematic illustration of the chimeric CDs construction strategy based on *Escherichia coli* IscS and human NFS1. (I) IscS, (II) NFS1, (III) chimeric CD EH-IscS, (IV) chimeric CD HE-IscS. Frames of the same colour represent the same domain.

**Figure 3:**
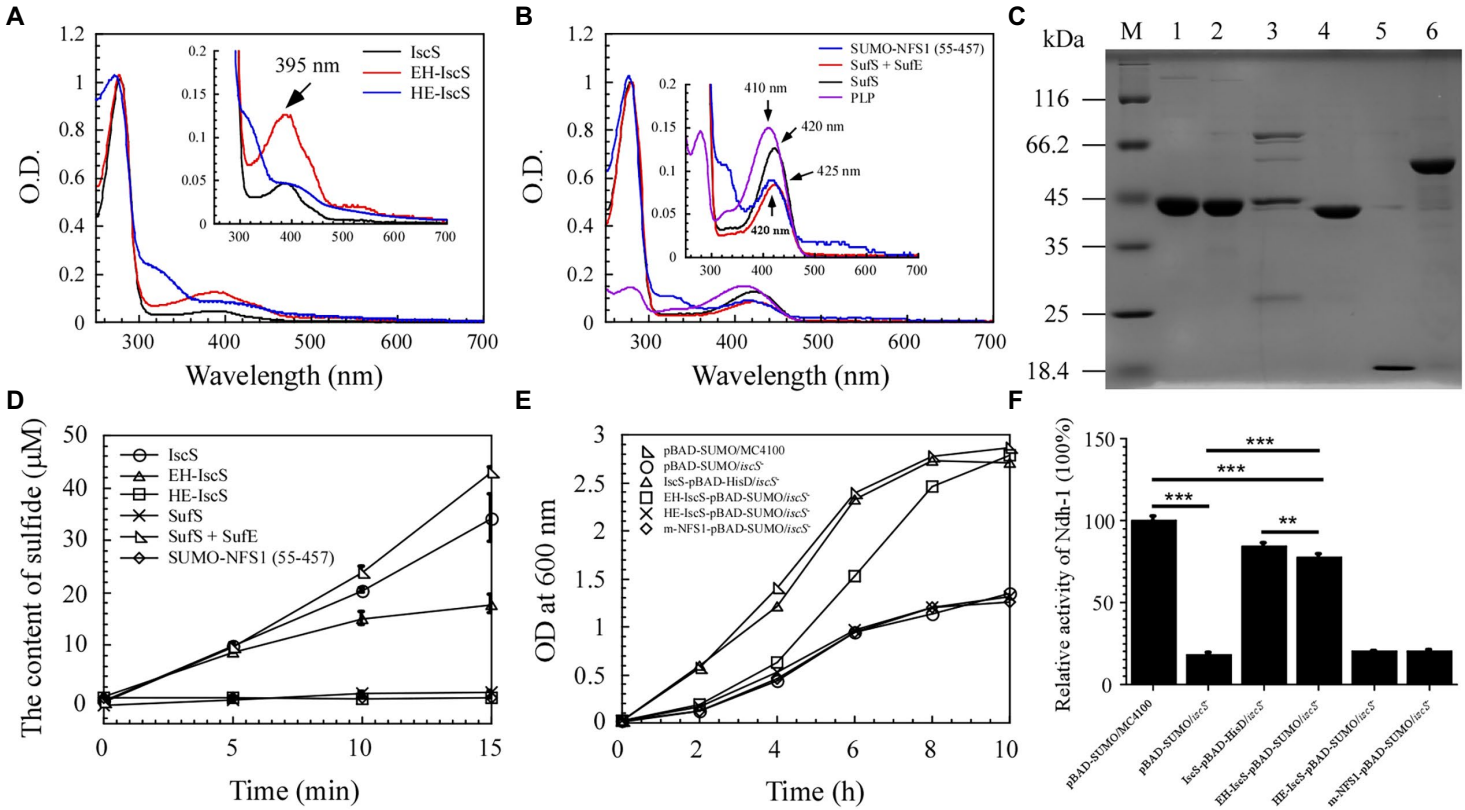
The chimeric CD EH-IscS exhibited desulphurising enzymatic activity, and it restored cell growth of the △*iscS* single mutant. **(A)** UV-visible spectra of purified IscS (25 μM), EH-IscS (29 μM), and HE-IscS (24 μM); **(B)** UV-visible absorption spectra of purified SUMO-NFS1 (55–457; 27 μM), SufS (21 μM), SufE (48 μM), and PLP (20 μM). The absorption peak at 395 nm (Figure 3A) or 420 nm (Figure 3B) indicates PLP contained in recombinant CDs. Inserts in Figures 3A,B are megascopic absorption spectra of PLP in recombinant CDs. All proteins were dissolved in buffer A containing 500 mM of NaCl and 20 mM of Tris–HCl (pH 8.0); **(C)** SDS-PAGE gel of purified recombinant CDs. Lane M, molecular weight markers; lane 1, purified IscS; lane 2, purified EH-IscS; lane 3, purified HE-IscS; lane 4, purified SufS; lane 5, purified SufE; lane 6, purified SUMO-NFS1 (55–457); **(D)** The CD activity of purified recombinant IscS, EH-IscS, HE-IscS, SUMO-NFS1(55–457), SufS, and SufS + SufE (5 μM) was measured using Siegel’s (Siegel, 1965) sulphide detection method; **(E)** Complementary role of chimeric CD on the △*iscS* mutant cell growth in liquid LB medium supplemented with 0.002% arabinose; **(F)** Effects of chimeric CD on the activity of NADH dehydrogenase I in the *Escherichia coli* △*iscS* mutant cells. At least three independent experiments were carried out for the growth curve and activity assay. The data are presented as the mean ± SD (*n* = 3). ** indicates *p* < 0.01, and *** indicates *p* < 0.001.

Several studies have indicated that recombinant preparation of human NFS1 is challenging (Cai et al., 2017). The majority of the interactions with PLP in NFS1 were similar to those of IscS (Figures 1, 2) as revealed by an analysis of active site residues. Nonetheless, NFS1 is only activated when [Acp]_2_:[ISD11]_2_:[NFS1]_2_ complexes are formed (Cory et al., 2017). Two types of chimeric CDs were constructed in this study to explore the binding domain of the red substance in IscS and why NFS1 alone was inactive. One strategy was to construct chimeric HE-IscS by fusing the N-terminal domain of NFS1 (amino acids 55–315) with the C-terminal domain of IscS (amino acids 264–404; Figure 2). As illustrated in Figure 3A, UV–visible absorption indicated that purified HE-IscS exhibited no distinct absorption peak at 395 nm. Moreover, HE-IscS activity almost disappeared (Figure 3D). Another approach was to construct chimeric EH-IscS by fusing the N-terminal domain of IscS (amino acids 1–263) and the C-terminal domain of NFS1 (amino acids 316–457; Figure 2). As shown in Figure 3A, UV–visible absorption indicated that purified EH-IscS and IscS proteins exhibited the same PLP absorption peak at 395 nm. Moreover, EH-IscS exhibited strong desulfurase activity (Figure 3D).

Purified NFS1 (55–457) and SufS absorption spectra exhibited maximum absorption at 420 nm, which resulted from the PLP cofactor (Figure 3B). While NFS1 (55–457) was yellow and inactive (Figure 3D). Upon SufE binding, the emission maximum for SufS shifted from 420 to 425 nm (Figure 3B). Purified SufS had no CD activity, but the presence of SufE significantly increased SufS activity, and SufS/SufE complex activity was even higher than that of IscS (Figure 3D). Similarly, the UV–visible absorption spectrum of PLP exhibited an absorption maximum at 410 nm (Figure 3B). As shown in Figure 3C, aside from HE-IscS (purity lower than 50%), all the other purified recombinant CDs displayed single bands on the SDS-PAGE gel. These results indicate that the N-terminus of CD is crucial for its activity, and the red intermediate is likely to be bound to the N-terminus of IscS.

Previous studies have shown that deletion of *iscS* reduces growth and lowers the activity of iron–sulphur cluster-dependent enzymes (2–50% of the wild-type) in *E. coli* (Schwartz et al., 2000; Rybniker et al., 2014). NADH quinone oxidoreductase (complex I) is essential for cellular energy metabolism, and NADH dehydrogenase I requires multiple iron–sulphur clusters to maintain its catalytic activity (Nakamaru-Ogiso et al., 2008). Although EH-IscS self-aggregated *in vitro* (Li et al., 2018), and its activity decreased gradually with prolonged incubation time compared with wild-type IscS (Figure 3D), our previous study indicated that using the SUMO tag as a fusion cofactor improved EH-IscS solubility and stability. Moreover, the SUMO-EH-IscS fusion protein had greater activity than the His-tagged EH-IscS control (Li et al., 2018). Therefore, these results suggest that chimeric SUMO-EH-IscS has the potential to treat mitochondrion-related rare diseases in humans caused by [Acp]_2_:[ISD11]_2_:[NFS1]_2_ complex mutations (Saha et al., 2015). Consistent with IscS, we found that introducing SUMO-EH-IscS into the *E. coli* △*iscS* single mutant significantly restored the cell growth and activity of NADH dehydrogenase I (Figures 3E,F).

### 3.2. Mutating the conservative active site residues in IscS leads to shifting the absorption peak of the PLP cofactor and the emergence of new absorption peaks

**Figure 4:**
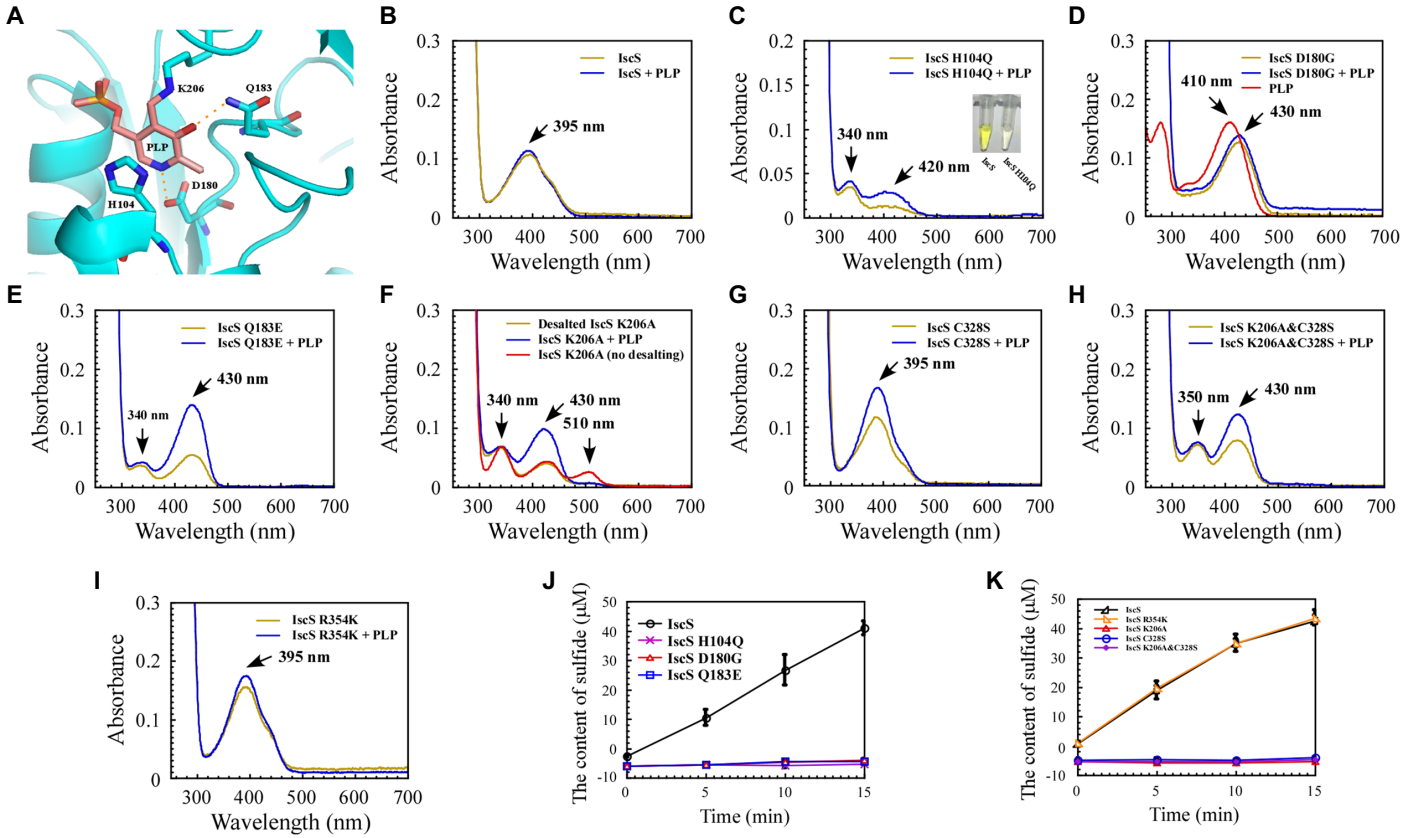
Mutation of the key residues of *Escherichia coli* IscS resulted in a set of intermediates generated *in vivo* during the CD reaction. **(A)** Active site structure of *E. coli* IscS. The PLP and PLP-binding residues are shown in the sticks model. The PLP cofactor makes a covalent link to Lys206. Polar interactions stabilising the position of PLP in the active site are indicated by orange dashed lines. This image was generated using PyMOL; **(B–I)** UV–visible spectral changes of purified wild-type IscS and IscS variants (H104Q, D180G, Q183E, K206A, C328S, K206A&C328S, R354K) are expressed in the *E. coli* wild-type cells, with or without PLP (100 μM). The insert in **(C)** is a photograph of IscS and IscS H104Q proteins purified from the *E. coli* wild-type cells. All proteins we obtained followed a protein purification protocol in which the solution (with or without exogenous PLP) is incubated for 30 min on ice as soon as the *E. coli* cells pass through an NS1001L2K high-pressure cell cracker, and they are purified by a Ni-agarose column (Qiagen co.) and then desalted by a gel filtration desalting column (SuperdexTM 75 10/300GL, GE). The protein concentration was calibrated to approximately 25 μM with buffer A. (No desalting in **F**) IscS K206A was measured by UV–visible spectra after being purified through a Ni-agarose column without desalting. Prepared overexpressed CDs always contain apoproteins; + PLP in **(B–I)** indicates that the purified IscS and IscS variants bind to exogenous PLP saturated *in vitro*; **(J,K)** Effects on the activity of wild-type *E. coli* IscS and IscS variants purified from *E. coli* wild-type cells. The protein concentration was calibrated to 5 μM with buffer A. CD activity of IscS and IscS variants was measured using Siegel’s sulphide detection method (Siegel, 1965). All data were obtained from three independent experiments.

Site-directed mutagenesis was used to trap reaction intermediates of the IscS enzyme in order to better understand its catalytic mechanism. Both, the active-site pocket (Figure 4A) and the active-site loop of IscS are highly conserved from prokaryotes to eukaryotes, as illustrated in Figures 1, 2. In general, purified IscS was yellow because of the presence of PLP cofactor (Figure 4C). Direct observation of the purified wild-type IscS and IscS variants (H104Q, Q183E, K206A, K206A, and C328S) revealed that the colour of the IscS variants was lighter, and the colour of IscS-H104Q was the lightest (Figure 4C), whereas the yellow colour of the IscS-D180G variant was significantly enhanced, reflecting that the active site in the N-terminal of IscS affected the binding of PLP (Figure 4). However, because the absorption peak of PLP drifts in the five IscS variants, measuring and calculating the relative content of PLP in the IscS variants based on their extinction coefficients is challenging.

The purified IscS showed a PLP absorption peak at 395 nm (Figure 4B). Interestingly, except for IscS-C328S and IscS-R354K (Figures 4G,I), the absorption peak at 395 nm disappeared for the other IscS variants, and new absorption peaks appeared. For instance, purified IscS-H104Q had two new absorption peaks at 340 and 420 nm (Figure 4C); purified IscS-D180G had a new absorption peak at 430 nm (Figure 4D); purified IscS-Q183E had two new absorption peaks at 340 and 430 nm (Figure 4E); purified IscS-K206A had three new absorption peaks at 340, 430, and 510 nm (Figure 4F); and purified IscS-K206A&C328S had two new absorption peaks at 350 and 430 nm (Figure 4H). It should be noted that the absorption peak at 510 nm of the red IscS K206A variant was not stable and disappeared once this variant was desalted by gel filtration (SuperdexTM 75 10/300GL, GE; Figure 4F), which is consistent with the results presented by Yang et al. (2015). These results demonstrated that the key active site residues have different roles in PLP binding to IscS, affecting the reaction progress and spectral characteristics of IscS.

Because the recombinant proteins are overexpressed while PLP synthesised by the host strain is limited, some of the purified proteins are apoproteins. We treated the cell lysates of IscS and IscS variations with or without PLP buffer (final concentration: 100 μM) before purifying the recombinant CDs to assess whether these new absorption peaks are likely to represent trapped catalytic intermediates of IscS. The results showed that when incubated with exogenous PLP, the absorption peak of purified IscS variants (H104Q, Q183E, K206A, K206A&C328S) at 420 nm/430 nm or IscS-C328S and IscS-R354K at 395 nm increased to varying degrees, except for IscS and IscS D180G (Figures 4B–I), whereas the absorption peak of PLP standard dissolved in buffer A was at 410 nm (Figure 4D). Furthermore, as shown in Figures 4J,K, most of the IscS variants lost desulfurase activity. However, compared with the wild-type IscS, IscS-R354K had full activity as IscS (Figure 4K). These results indicate that the key active site residues (His104, Asp180, Gln183, Lys206, and Cys328) of IscS are indispensable for its activity. Moreover, different active site residues may play different roles in the enzymatic reaction of IscS.

In conclusion, only chimeric EH-IscS constructed by fusing the N-terminal domain of *E. coli* IscS with the C-terminus of human NFS1 was active in the two types of chimeric CD (Figure 3D). Furthermore, site-directed mutagenesis of IscS at the Asp180 and Gln183 sites that form hydrogen bonds with PLP (Figure 4A) resulted in an absorption peak similar to that of NFS1 (Figure 3B) and loss of enzyme activity (Figures 4D,E,J). These results suggest that the N-terminus of IscS, especially the hydrogen bonds interacting with PLP, is crucial for its activity. Furthermore, we speculate that the absorption peak of IscS variants at 420 nm or 430 nm is the absorption peak of PLP after it binds to the apoproteins of IscS variants. On the other hand, these results also indirectly indicate that the absorption peaks at 340 nm and 350 nm correspond to cysteine-PLP ketimine or cysteine-PLP aldimine intermediates (Nakamura et al., 2020).

### 3.3. Kinetic analysis of the enzymatic reaction process of IscS

**Figure 5:**
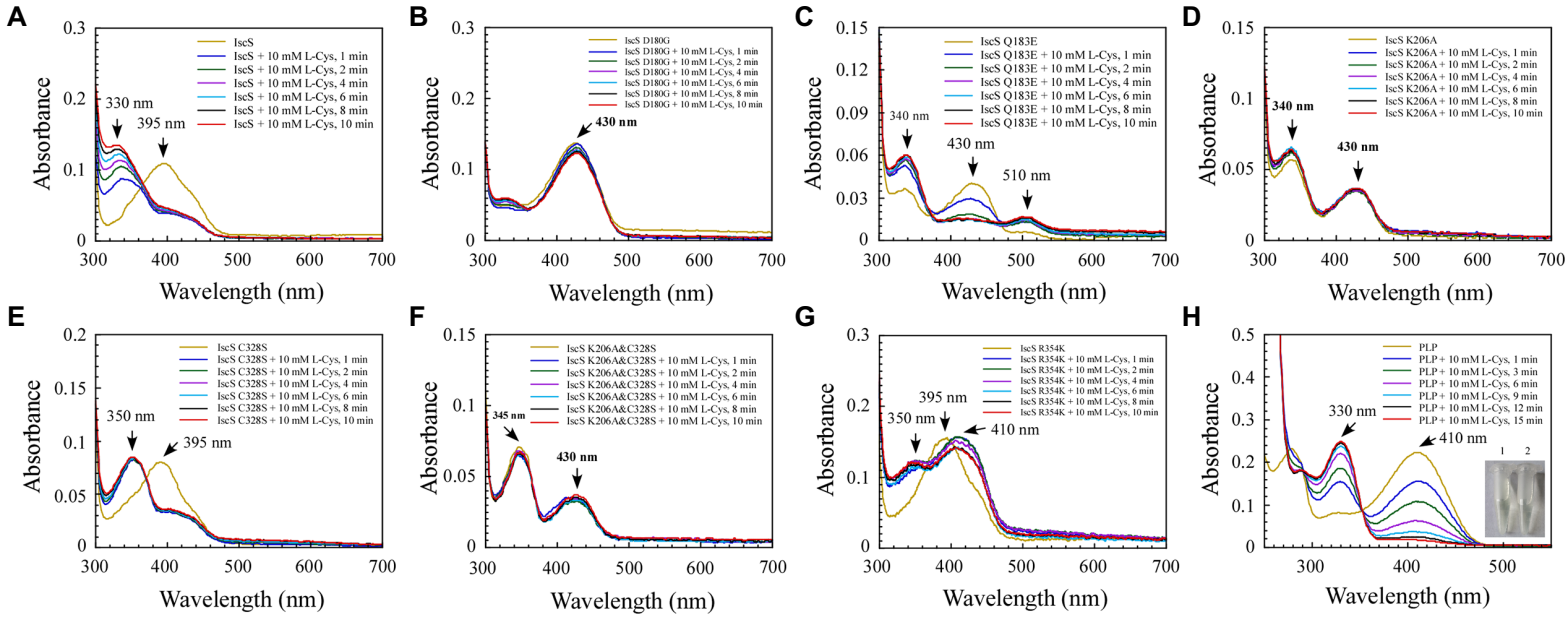
The reaction progress of *Escherichia coli* IscS is dynamically displayed in the experiment of incubating and binding wild-type IscS (IscS-WT) and its variants with L-cysteine (substrate) *in vitro*. **(A–G)** UV–visible spectral changes of IscS-WT and its variants (25 μM) upon the addition of 10 mM of L-cysteine. Arrows show changes in absorption peaks at these wavelengths. It is noted that CD incubation with 10 mM of L-cysteine at room temperature for 10 min results in the decline or disappearance of the absorption peak at 395 nm or 430 nm, whereas a new set of absorption peaks correspond with the appearance of a series of reactional intermediates in an unstable manner; **(H)** The external ketimine or aldimine intermediates produced by the *in vitro* binding between PLP (50 μM) and L-cysteine (substrate) were used as the positive controls. The insets show photographs of PLP solution (1) and its reaction with L-cysteine (10 mM) after 15 min (2). The data are representative of three independent experiments.

**Figure 6:**
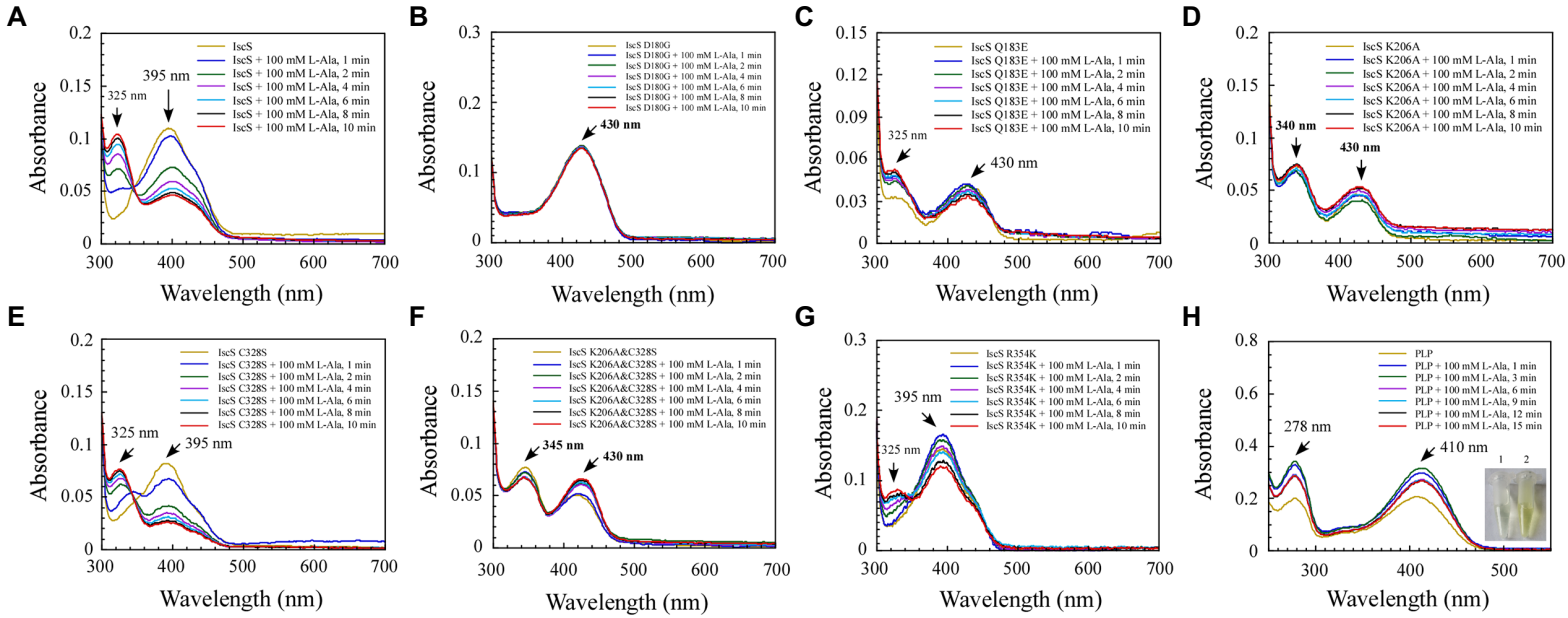
Changes in the absorption spectrum of IscS-WT and its variants associated with the formation of reaction with the L-alanine (product) *in vitro*. **(A–G)** Time-dependent spectral analysis of L-alanine binding to IscS-WT and its variants; **(H)** UV–visible spectral changes of 50 μM PLP upon the addition of 100 mM L-alanine (product). The insets show photographs of PLP solution (1) and its reaction with L-alanine after 15 min (2). The data are representative of three independent experiments.

To enable direct observation of intermediates of the CD reaction, we incubated 10 mM excess L-cysteine or 100 mM excess L-alanine with wild-type IscS and IscS variants, respectively. As shown in Figure 5A, upon incubating wild-type IscS with excess L-cysteine, its absorption peak at 395 nm disappeared almost immediately, and a new absorption peak corresponding to an unstable reaction intermediate appeared at approximately 330–340 nm. However, L-alanine addition resulted in a decrease in absorption at 395 nm and a concomitant increase in absorption at 325 nm. The reaction rate was also significantly lower than that of L-cysteine (Figure 6A).

Among the six IscS variants, upon incubating the IscS Q183E variant with L-cysteine, a new absorption peak appeared immediately at 510 nm, but this new absorption peak showed no time accumulation effect (Figure 5C). The IscS C328S variant exhibited a similar phenomenon to wild-type IscS, but when incubated with L-cysteine, the reaction intermediate absorbing at 350 nm was stable, and the magnitude of its drift over time was very low (Figure 5E). The time-dependent spectrum of the IscS C328S variant reacting with L-alanine was consistent with that of wild-type IscS, except for the appearance of a transient absorption peak at 345 nm (Figure 6E). Interestingly, when the IscS R354K variant was incubated with L-cysteine or L-alanine, the enzymatic reaction rate was significantly lower than that of the wild-type IscS, and the spectral absorption peak of PLP shifted significantly (Figures 5G, 6G). It is worth noting that when the purified IscS variants (D180G, K206A, K206A&C328S) were incubated with L-cysteine or L-alanine, the time-dependent UV–visible spectra of these three IscS variants changed only slightly or was almost unaffected compared with the wild-type IscS (Figures 5, 6).

Furthermore, the formation of the reaction intermediates in SUMO-EH-IscS, SUMO-NFS1 (55–457), and SufS were investigated. Similar to wild-type IscS, incubation of SUMO-EH-IscS with L-cysteine resulted in a decrease in the absorbance of PLP, whereas a new absorption peak corresponding to an unstable reaction intermediate appeared at approximately 330–340 nm (Supplementary Figure S1A). Interestingly, because SufS and SUMO-NFS1 (55–457) were inactive, although incubation with excess L-cysteine led to a rapid decrease in the absorption peak of PLP, the absorption peaks of the enzymatic reaction intermediates generated were all fixed at 340 (Supplementary Figure S1B) and 330 nm (Supplementary Figure S1D). However, when SufS and SufE were simultaneously incubated with excess L-cysteine, the absorption peaks of the enzymatic reaction intermediates shifted during the reaction process (Supplementary Figure S1C). Similarly, PLP could react directly with L-cysteine or L-alanine *in vitro*, resulting in an increase in absorbance at 330 or 278 nm, and the newly formed absorption peak at 330 nm was stable and had a time-accumulation effect (Figures 5H, 6H). Interestingly, when PLP was incubated with L-cysteine, the absorption peak at 410 nm gradually decreased and almost disappeared, and the PLP solution changed from light yellow to colourless (Figure 5H). However, when PLP was incubated with L-alanine, the absorption peak at 410 nm first increased and then decreased, and was significantly higher than the absorption peak of PLP before the reaction, especially when the PLP solution changed from light yellow to bright yellow (Figure 6H). These results indicate that the newly formed absorption peak of the IscS variants *in vitro* is very similar to the absorption peak of the IscS variants accumulated in *E. coli* cells (Figures 4C–H). This further indicates that the absorption peaks at 325–355 nm shown in the IscS variants could correspond to different intermediates during the enzymatic reaction of IscS and that the intermediates may be conjugated to the pyridoxal ring, which can prevent the new L-cysteine substrate or L-alanine product from binding to PLP.

### 3.4. Formation of red intermediate in wild-type IscS and IscS variants (Q183E, K206A) *in vitro*

**Figure 7:**
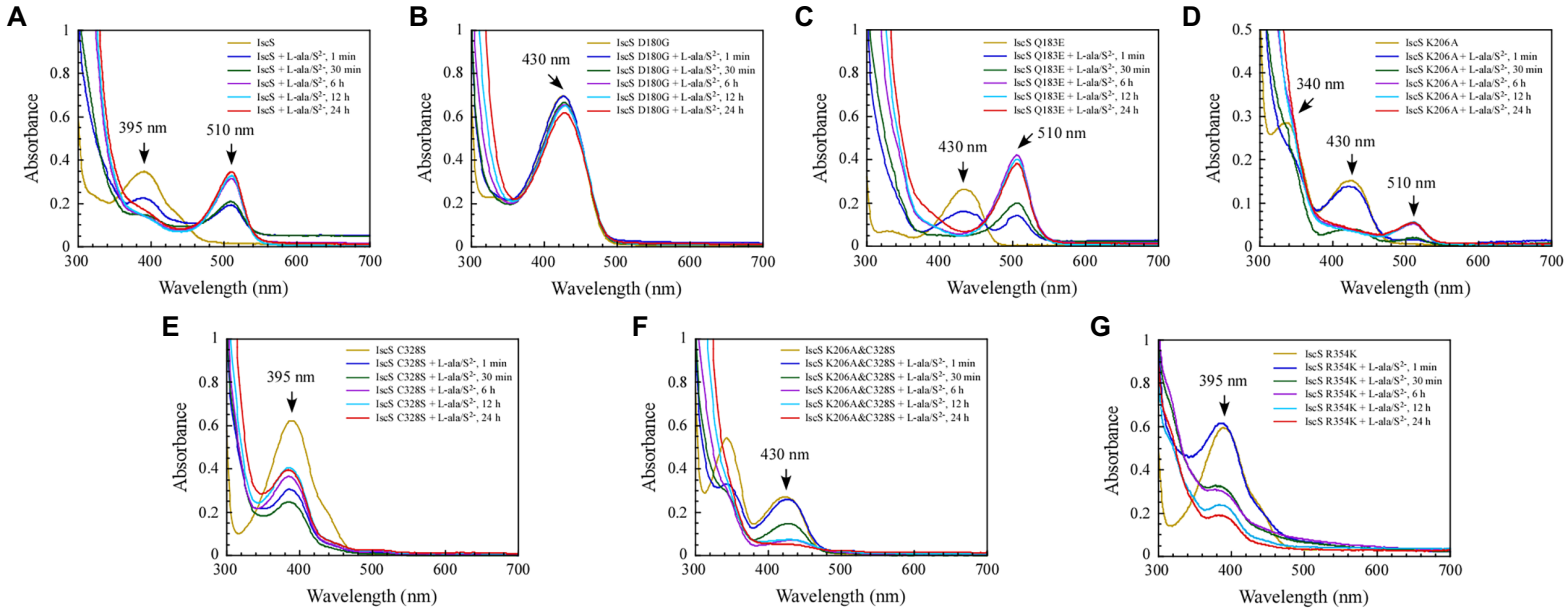
The formation of a red intermediate in wild-type IscS and IscS variants (Q183E, K206A) *in vitro*. **(A–G)** Purified IscS (100 μM) and IscS variants (H104Q, D180G, Q183E, K206A, C328S, K206A&C328S, R354K; 100 μM) were incubated with L-alanine (100 mM) and Na_2_S (100 mM) in Tris (120 mM, pH 8.0) buffer at room temperature. The UV–visible absorption spectra were recorded at 1 min, 30 min, 6 h, 12 h, and 24 h. The data shown are the results of three independent experiments.

Previous studies (Yang et al., 2015) have shown that when wild-type IscS is incubated with excess L-alanine and sulphide under aerobic conditions, red IscS gradually forms *in vitro* and has an absorption peak at 510 nm (Figure 7A), which is the same as that of red IscS purified from *E. coli* iscA/sufA mutant cells after oxidation with H_2_O_2_ (Yang et al., 2015). Interestingly, the absorption peak at 510 nm for red IscS was very close to the absorption peak at 506 nm of the proposed alanine quinonoid intermediate in CD0387 from *Synechocystis* sp. PCC 6803 (Behshad and Bollinger, 2009). Interestingly, upon the reaction of IscS Q183E or IscS K206A variants with L-alanine and sulphide *in vitro*, a new absorption peak appeared at 510 nm (Figures 7C,D). However, unlike wild-type IscS or IscS Q183E incubated with L-alanine and sulphide, the absorption peak at 510 nm accumulated very slowly in the IscS K206A variant, and the peak shape amplitude was low (Figure 7D). Unexpectedly, the IscS R354K variant, which has the same activity as wild-type IscS, reacted with L-alanine and sulphide for 24 h and did not produce an absorption peak at 510 nm (Figure 7G). In addition, the IscS D180G, IscS C328S, and IscS K206A&C328S variants did not generate a new absorption peak at 510 nm when reacting with L-alanine and sulphide (Figures 7B,E,F). These *in vivo* and *in vitro* (Figures 4F, 5C) results suggested that the absorption peak at 510 nm could be due to the quinonoid intermediates produced by wild-type IscS and its mutants (Q183E and K206A) during the enzymatic reaction.

### 3.5. Isolation and identification of suspected enzymatic reaction intermediates from IscS variants

**Figure 8:**
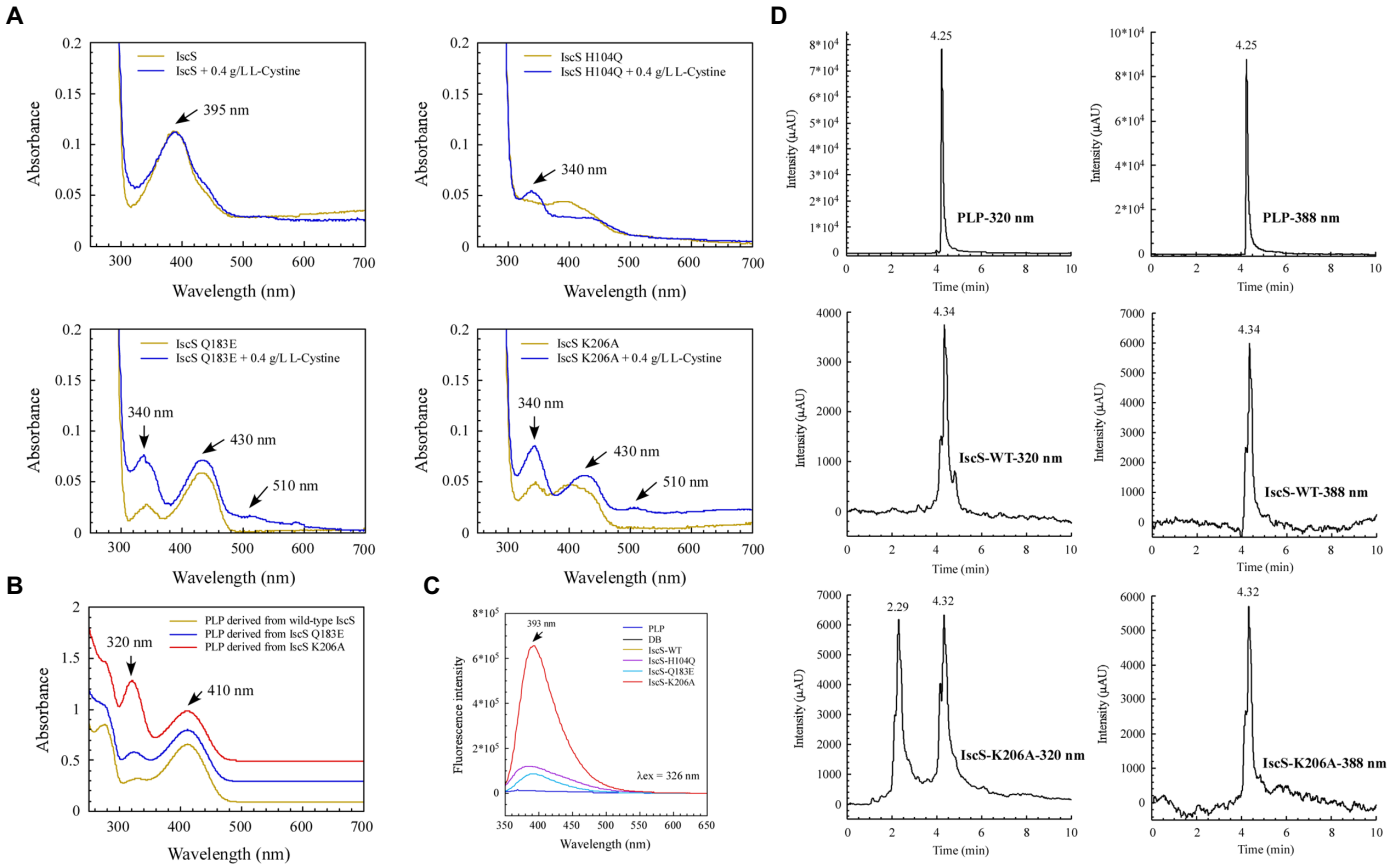
Isolation and identification of suspected intermediates from the enzymatic reaction of IscS. **(A)** UV–visible spectra of purified IscS-WT and its variants (25 μM) are expressed in the BL21(DE3) cells, with or without L-cystine. When the *Escherichia coli* cells grown in LB media reached OD_600_ of 0.6, the recombinant CDs were induced with 0.02% arabinose for 3 h at 37°C. Then, the cells were collected by centrifugation and resuspended in an equal volume of M9 basal media. Chloramphenicol (34 μg/ml) was added, with or without L-cystine (0.4 g/L). After incubation at 37°C for 3 h, the cells were collected for purified recombinant CDs; **(B)** UV–visible spectra of the supernatant of desalted IscS-WT and IscS variants after heating at 100°C for 10 min; **(C)** Fluorescence spectra of PLP and its derivatives derived from IscS-WT and IscS variants. The sample preparation method was the same as that of **(B)**; the concentration was about 100 mM. The excitation wavelength was set at 326 nm; **(D)** HPLC profiles of PLP standard (100 μM) and PLP derived from wild-type IscS and IscS K206A. Chromatography was performed using an Agilent 1260 Infinity HPLC system. The chromatographic separation was performed on an Ecosil C18-AQ PLUS column (4.6 mm × 250 mm, 5 μm) with a mobile phase consisting of acetonitrile and ammonium acetate buffer (10 mM, pH 5.6; 30:70, v/v) at a flow rate of 0.5 ml/min. The temperature of the column was maintained at 25°C. The injection volume was 10 μl. The UV detection wavelength was set at 320 and 388 nm, respectively.

We sought to further demonstrate that the IscS variants generate new absorption peaks from the adducts formed by L-cysteine and PLP. Of note, L-cysteine is easily oxidised *in vitro*. Therefore, in M9 basal medium, we incubated *E. coli* BL21(DE3) cells overexpressing IscS and its variants (H104Q, Q183E, and K206A) with or without L-cysteine. UV–visible spectral analysis showed that absorption peaks at 340 nm and 510 nm were indeed derived from the intermediates generated by the reaction between the L-cysteine substrate and PLP catalysed by IscS variants (Figure 8A).

Interestingly, after heating the IscS, IscS Q183E, and IscS K206A variants at 100°C for 10 min, the intermediates generated by the L-cysteine reaction with PLP were stable, and the supernatant of the IscS K206A variant had the highest absorption peak at 320 nm (Figure 8B). Moreover, the spectrum of the wild-type IscS supernatant was consistent with that of the PLP standard (Figure 4D). Unfortunately, heating at 100°C resulted in the disappearance of the red IscS colour, and the UV–visible spectrum of the supernatant was consistent with that of the wild-type IscS (data not shown). Furthermore, fluorescence spectral analysis showed that neither the PLP standard nor PLP derived from wild-type IscS could emit fluorescence under excitation light at 326 nm (Figure 8C). However, the intermediates from the IscS K206A variant had a maximum emission spectrum at 393 nm (Figure 8C), and the emission spectral intensity was consistent with that shown in Figure 8B.

We speculated that the simultaneous absorption peaks at 320 and 410 nm of the intermediate from the IscS K206A variant (Figure 8B) may be caused by the mixture of PLP and cysteine-PLP adducts, and therefore isolated this intermediate using HPLC. As shown in Figure 8D, the retention time of PLP was 4.25 min under dual-wavelength detection at 320 and 388 nm. The retention times of PLP derived from wild-type IscS and the IscS K206A variant were consistent with that of the PLP standard. Interestingly, at a detection wavelength of 320 nm, the protein supernatant of the IscS K206A variant showed a new peak at a retention time of 2.29 min, indicating that the intermediate was successfully separated. Furthermore, the UPLC-MS identification results were consistent with the HPLC results. As shown in Supplementary Figures S2A,B, the molecular weight of 248.1 and 351 Da that correspond to PLP standard and PLP-L-cysteine aldimine was derived from the reaction product of PLP and L-cysteine, respectively. These results suggest that further isolation and identification of enzymatic reaction intermediates from IscS variants (H104Q, Q183E, K206A, and K206A&C328S) can be performed by UPLC-MS/MS in the future. In conclusion, these results demonstrate that the enzymatic reaction of L-cysteine catalysed by IscS is a multi-step process, and mutation of its key active site residues facilitates the trapping of a variety of reaction intermediates.

## 4. Discussion

CDs (EC 2.8.1.7) can be categorised as type I and type II enzymes depending on their structure and reactivity. Type I CDs are similar to NifS and IscS, whereas type II CDs include SufS-like and cysteine sulfinate desulfinase A (CsdA)-like proteins (Dunkle et al., 2019; Das et al., 2021). CDs use the cofactor PLP to extract sulphur from free L-cysteine, resulting in the production of L-alanine and persulfide. Despite considerable progress in recent years, the stepwise mechanism by which this PLP-dependent enzyme operates remains unclear (Blahut et al., 2019; Nakamura et al., 2020). Therefore, to study the binding properties of PLP, the formation process of intermediates, and the physiological significance of accumulated red IscS, we selected several (active-site) residues associated with PLP binding to carry out site-directed mutagenesis. Moreover, UV–visible absorption spectra analysis, structural analysis, HPLC and UPLC-MS spectra analysis, and activity determination of the purified wild-type IscS, IscS variants, and red IscS were conducted.

**Figure 9:**
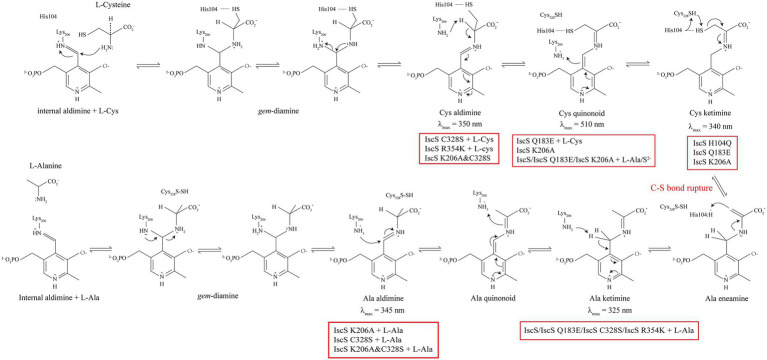
Proposed intermediates of *Escherichia coli* IscS formed in the reaction coordinate. The red box indicates the IscS variants or the reaction that can generate the corresponding intermediate products.

In this study, site-directed mutations at the six sites of His104, Asp180, Gln183, Lys206, Cys328, and Arg354 in IscS were conducted. New absorption peaks were found in the four mutant proteins, and the activity of all IscS variants was also considerably changed, except for that of the IscS R354K variant (Figure 4). Interestingly, there was a new absorption peak at *λ*_max_ approximately 340 nm for purified IscS-H104Q, IscS-Q183E, and IscS-K206A (Figures 4C–F, 8A). This was identical to the absorption peak in the intermediate of Cys-ketimine (λmax approximately 340 nm), which is formed in the process of CD0387 catalysis (Behshad and Bollinger, 2009). Nakamura et al. (2020) suggested that the conserved histidine adjacent to PLP stabilises the thiol group of the PLP-L-cysteine external aldimine through polar interactions. This interaction orientates the thiol group for subsequent nucleophilic attack by a conserved cysteine residue on the catalytic loop in the Cys-ketimine state, which is formed from Cys-aldimine (Figure 9). Therefore, we speculated that after the mutation of His104, the protonation process was inhibited and the catalytic reaction that remained before the C-S bond was broken, which in turn resulted in the accumulation of the Cys-ketimine intermediate (Figure 9). This also indicated that the π–π stacking interaction between His104 and the pyridoxal ring of PLP is essential for the stable binding of PLP to the active site pocket (Figure 4C).

Consistent with the above, the mutation of Gln183 caused the absorption peak of PLP to shift from 395 to 430 nm (Figure 4E), indicating that the position of PLP in the active-site pocket was shifted, which could disrupt the polar interaction between His104 and the thiol group, leading to the accumulation of the Cys-ketimine intermediate (Figures 4E, 5C, 9). Moreover, a hydrogen bond is formed between the Gln183 residue and phenolate oxygen of PLP, and these intermolecular forces are associated with PLP proton transfer (Cupp-Vickery et al., 2003). Therefore, the Gln183 site mutation also disrupted the rapid equilibrium between Cys-aldimine and Cys-ketimine, which was conducive to the accumulation of the Cys-quinonoid intermediate (Figures 5C, 7C, 9). Although disruption of the polar interaction of Asp180 with the pyridine N1 of PLP also resulted in a shift in the absorption peak of PLP from 395 nm to 430 nm (Figure 4D), the substrate/product-binding assay showed that the absorption peak of IscS D180G at 430 nm did not change significantly (Figures 5B, 6B). Thus, it is suggested that Asp180 is an essential site for the formation of PLP-substrate and PLP-product external adducts, which could be because L-cysteine and L-alanine cannot effectively contact PLP after PLP is shifted in the active-site pocket.

Cupp-Vickery et al. (2003) showed that the PLP cofactor is anchored in the active-site pocket by the formation of an internal aldimine Schiff base with Lys206. Unexpectedly, a certain amount of the PLP cofactor was still bound in the active site pocket after the Lys206 site mutation; however, similar to SufS (420 nm; Figure 3B), the absorption peak of IscS K206A also shifted to 430 nm (Figure 4F). Therefore, IscS K206A, like the IscS H104Q and IscS Q183E variants, has no desulfurase activity (Figure 4K), and the enzymatic reaction stops C-S bond cleavage (Figure 9). Consistent with this, non-desalted IscS K206A showed new absorption peaks at 340 and 510 nm (Figure 4F). Blahut et al. (2019) showed that Lys-226 of SufS can act as a general base to deprotonate the Cα position to form the Cys-quinonoid intermediate. Then, Lys-226 might transfer the proton to the C4’ position of PLP to form Cys-ketimine. It has also been suggested that Cys-364 of SufS is essential for positioning the Cys-aldimine for Cα deprotonation and that His123 of SufS is responsible for protonation at the C4’ position during the formation of Cys-ketimine from Cys-quinonoid (Behshad and Bollinger, 2009; Blahut et al., 2019). We found that a stable PLP-L-cysteine adduct (330 nm) was formed immediately upon incubation of PLP (410 nm) with L-cysteine *in vitro* (Figure 5H). Therefore, the reaction between PLP and L-cysteine is a natural reaction in the IscS K206A variant. Moreover, the interaction between PLP, His104, and other active site residues still exists in the IscS K206A variant. Based on these results, we speculate that Cys328 may replace His104 to position the Cys-aldimine for Cα deprotonation; His104 may replace Lys206 to deprotonate the Cα position of Cys-aldimine to form the Cys-quinonoid intermediate and transfer the proton to the C4’ position of Cys-quinonoid to form Cys-ketimine in the IscS K206A variant, to also partially enable the enzymatic reaction (Figures 4F,H), but at a very low rate.

As shown in Figures 4G,H, the purified IscS C328S variant only had an absorption peak at 395 nm, whereas the purified IscS K206A&C328S variant had absorption peaks at 350 and 430 nm, respectively. Furthermore, when IscS C328S was incubated with excess L-cysteine, a new stable absorption peak immediately formed at 350 nm and had no time-accumulating effect (Figure 5E). Therefore, we speculated that the prerequisite for the reaction of L-cysteine with the internal aldimine to form the Cys-ketimine intermediate was the presence of an active Cys328 site in the catalytic loop (Figure 9). In conclusion, we speculate that the stable absorption peak at 350 nm of IscS C328S reacted with L-cysteine, and the IscS K206A&C328S variant was attributed to Cys-aldimine intermediates (Figures 4H, 5E, 9). The absorption peaks at 340 nm and 510 nm of the IscS K206A variant were attributed to Cys-ketimine and Cys-quinonoid intermediates (Figure 9). Additionally, HPLC was used to successfully separate the two substances with absorption peaks at 340 and 430 nm in the IscS K206A variant (Figure 8D).

Consistent with our findings, Blahut et al. (2019) showed that SufS H123A or SufS C364A crystals incubated with L-cysteine possessed electron density for a Cys-ketimine or Cys-aldimine enzymatic intermediate covalently bound to PLP. Patra and Barondeau (2019) monitored three key intermediates – Cys-aldimine (410 nm), Cys-quinonoid (508 nm), and Cys-ketimine (340 nm) – generated during human NFS1 enzymatic reactions using stopped-flow kinetics. As shown in Figure 6, when IscS and its variants were incubated with excess L-alanine substrate, the reversible enzymatic reactions remained in the intermediate states of Ala-aldimine (345 nm; Figures 6E,F) or Ala-ketimine (325 nm; Figures 6A,C,E). Furthermore, when IscS and its variants were incubated with excess L-alanine and Na2S, only IscS, IscS Q183E, and IscS K206A accumulated Cys-quinonoid intermediates (Figure 7). These results suggest that Gln183 and Lys206 play key roles in the protonation/deprotonation and proton transfer of Cys-quinonoid, respectively. In addition, Blahut et al. (2019) showed that Arg359 of SufS changes to assist in positioning the Cys-aldimine for Cα-H bond cleavage. Consistent with this, the intermediates generated by the reaction of IscS R354K with L-cysteine remained in the Cys-aldimine state (Figure 5G). However, in the presence of the reducing agent DTT, the desulfurase activity of the IscS R354K variant was almost identical to that of the wild-type IscS (Figure 4K), whereas the reaction of IscS R354K with excess L-alanine and Na2S did not lead to the accumulation of red Cys-quinonoid (Figure 7G). Furthermore, IscS R354K reacted slowly with excess L-cysteine or L-alanine (Figures 5G, 6G). These results suggest that Arg354 in IscS accelerates the deprotonation of the Cys-aldimine/Ala-aldimine intermediate, which prompts its conversion to the Cys-quinonoid/Ala-quinonoid species, but it is not necessary for the enzymatic reaction to proceed. Taken together, these results enabled us to better understand the detailed mechanism of the desulfurase reaction catalysed by IscS (Figure 9).

Sulphur (S) is an essential element for *E. coli* cells and is usually mobilised from L-cysteine by IscS (Schwartz et al., 2000). As shown in Figures 4–8, we have demonstrated that the red substance of red IscS with the 510 nm absorption peak is Cys-quinonoid, and is probably derived from the cysteine-PLP adduct, whereas the red substance with the 528 nm absorption peak is presumed to be derived from Cys-quinonoid. However, the specific reaction mechanism is still unclear. Interestingly, as depicted in Figures 5D,F, 6D,F, the accumulation of Cys-ketimine and Cys-aldimine in the IscS K206A and IscS K206A&C328S variants avoids the competitive binding of new substrates (L-cysteine) and products (L-alanine) to the pyridoxal ring. Similar to our findings, Karsten and Cook (2009) reported that the reaction of enamine with PLP in L-serine-glyoxylate aminotransferase from Hyphomicrobium methylovorum generates a stable, highly conjugated quinonoid intermediate with an absorption peak at 521 nm. Our previous work also showed that the absorption peak at 528 nm of red IscS quickly shifted to 510 nm when red IscS was oxidised by H_2_O_2_, whereas the absorption peak at 395 nm for red IscS and wild-type IscS did not change. In particular, although the absorption peak at 528 nm of red IscS was not affected by sodium borohydride, which is a strong reducing reagent, its desulphurisation enzyme activity completely disappeared. However, oxidised red IscS remains largely active, but lacks the absorption peak at 528 nm (Yang et al., 2015). Based on these results, we speculate that the physiological significance of red IscS (528 nm) accumulation in *E. coli* cells could be a new way of regulating enzyme activity and that the biosynthesis of iron–sulphur clusters will be affected. As shown in Supplementary Figures S3A–D, under the premise that the amplitudes of the PLP absorption peak at 395 nm for the equimolar concentrations of the red and yellow CDs are in agreement, the activity of the red CDs was significantly lower than that of the yellow CDs, and the activity was inversely proportional to the amplitude of the absorbance peak at 528 nm. Further studies showed that the speed of assembly of the iron–sulphur cluster in IscU regulated by red IscS was lower than that of yellow IscS *in vitro* (Supplementary Figures S3E,F). In conclusion, these results suggest that IscS regulates its activity by accumulating red intermediates (528 nm) and then regulating iron–sulphur cluster biosynthesis. This might be an active physiologically protective behaviour for cells to adapt to stress conditions (such as a deficiency of accessible iron).

Corresponding to its physiological importance, the CD also influences the pathogenesis of pathogenic microorganisms. This is primarily related to how pathogenic CD mitigates drug-induced toxicity by manipulating cellular physiology and evades various host-derived oxidative stresses (Kohanski et al., 2007; Rybniker et al., 2014; Giordano et al., 2018; Das et al., 2021). For example, deletion of the *iscS* gene makes the human pathogen *Mycobacterium tuberculosis* hypersensitive to H_2_O_2_, whereas the overexpressing strain is more resistant to oxidative stress, implying a protective role (Rybniker et al., 2014). However, the *E. coli* △*iscS* mutant is resistant to antibiotics because the depleted iron–sulphur cluster pools impair Fenton-mediated •OH radicals during antibiotic exposure (Kohanski et al., 2007; Ezraty et al., 2013). Additionally, Alvarez et al. (2017) showed that NFS1 is overexpressed in metastatic or primary lung tumours. For example, lung adenocarcinomas select for overexpression of NFS1, which confers resistance to high oxygen tension and protects cells from ferroptosis in response to oxidative damage. Our previous work also showed that the mRNA and protein levels of iron–sulphur cluster biogenesis factor ISD11 were upregulated in hepatocellular carcinoma and that it could be a novel prognostic biomarker and molecular target for hepatocellular carcinoma therapy. Therefore, a comprehensive elucidation of the role of conserved active site residues in the CD reaction and the molecular mechanism by which CD manipulates its activity through the accumulation of the red quinonoid intermediate (528 nm) may contribute to its application in inhibiting pathogenic microorganisms and treating human diseases.

## Data availability statement

The original contributions presented in the study are included in the article/Supplementary material, further inquiries can be directed to the corresponding authors.

## Author contributions

YP: conceptualisation, data curation, formal analysis, software, supervision, investigation, methodology, and writing—original draft. JW, XG, MJ, and LZ: investigation and methodology. XW, FL, XXi, XR, and QS: investigation. TX and WW: writing, review, and editing. ML and XXu: software and formal analysis. JLy: funding acquisition and project administration. JLi: funding acquisition, project administration, and software. GT: conceptualisation, supervision, funding acquisition, project administration, and writing—review and editing. All authors contributed to the article and approved the submitted version.

## Funding

This study was supported by the Chinese National Natural Science Foundation of China Grants (Nos. 81671124 and 31870775) and the Key Discipline of Zhejiang Province in Medical Technology (first class, category A).

## Conflict of interest

Authors YP and XXi were employed by Hunan Animal Pharmaceutical Company, Hunan Agricultural Group Company, Hunan Agricultural Development & Investment Group Company, Wangcheng Economic and Technological Development Zone.

The remaining authors declare that the research was conducted in the absence of any commercial or financial relationships that could be construed as a potential conflict of interest.

## Publisher’s note

All claims expressed in this article are solely those of the authors and do not necessarily represent those of their affiliated organizations, or those of the publisher, the editors and the reviewers. Any product that may be evaluated in this article, or claim that may be made by its manufacturer, is not guaranteed or endorsed by the publisher.

## Footnotes

^1^ http://biotools.nubic.northwestern.edu/proteincalc.html
