# Supplementary material for: Roles of conserved active site residues in the IscS cysteine desulfurase reaction
Source: Front Microbiol. 2023 Feb 16;14:1084205. doi: 10.3389/fmicb.2023.1084205 (PMC9978102; doi:10.3389/fmicb.2023.1084205)
Supplement: Supplementary file 1 [file Data_Sheet_1.docx]

Supplementary Material

Roles of conserved active site residues in the IscS cysteine desulfurase reaction

Yilin Pang^1,2#^, Jing Wang^1#^, Xueping Gao^1#^, Mengyao Jiang^1#^, Lifei Zhu^1^, Feng Liang^1,4^, Mengxiang Liang^1^, Xiaolin Wu^1^, Xianxian Xu^1^, Xiaojun Ren^1,3^, Ting Xie^1^, Wu Wang^1^, Qianqian Sun^1^, Xiaojun Xiong^2^, Jianxin Lyu^1,3*^, Jianghui Li^1*^, Guoqiang Tan^1*^

*** Correspondence:** Jianxin Lyu, jxlu313@163.com, or Jianghui Li, lijianghui1980@126.com, or Guoqiang Tan, tgq@wmu.edu.cn.

# Supplementary Tables

**Supplementary Table S1** Bacterial strains and plasmids.

| **Strain or plasmid** | **Characteristics** | **Source or reference** |
| --- | --- | --- |
| *Escherichia.coli* strains |  |  |
| BL21(DE3) | F^-^ *omp*T *hsd*S(*r*_B_^-^ *m*_B_^-^) *gal* *dcm lon* (DE3) | CGMCC |
| DH5α | F^-^, λ^-^, *endA*1, *hsdR*17, *hsdM*^+^, *supE44*, *thi*1, *recA*1, *gyrA*96, *relA*1, Δ(*argF*, *lacZYA*), U169, φ80d, Δ(*lacZ*), M15 | CGMCC |
| MC4100 | F^-^, *araD139*△*(araF-lac)*, *U169*, *rspL150*, *relA1*, *flbB530*, *fruA25*, *deoC1*, *pstF25* | Prof. HG. Ding, Louisiana State University, USA |
| △*iscS* | MC4100 △*isc* | Wenzhou Medical University |
|  |  |  |
| Plasmids |  |  |
| pBAD/HisD | Amp^r^, Bacterial expression vector, *ara*BAD promoter, adds N-terminal His tag | Wenzhou Medical University |
| pBAD/His SUMO | Amp^r^, Bacterial expression vector, *ara*BAD promoter, adds N-terminal His tag and SUMO fusion tag | Wenzhou Medical University |
| pCold I | Amp^r^, Bacterial expression vector, Cold-shock expression vector, *csp*A promoter, adds N-terminal His tag | Takara |
| pCold TF | Amp^r^, Bacterial expression vector, Cold-shock expression vector, *csp*A promoter, adds N-terminal His tag and trigger factor tag | Takara |
| pCold-SUMOa | Amp^r^, Bacterial expression vector, Cold-shock expression vector, *csp*A promoter, adds N-terminal His tag and SUMO fusion tag | Wenzhou Medical University |
| IscS-pBAD/HisD | Amp^r^, the *E. coli* cysteine desulfurase IscS cloned into pBAD/HisD via *Kpn* I | This study |
| IscS-pBAD/*Myc*-HisC | Amp^r^, the *E. coli* cysteine desulfurase IscS cloned into pBAD/*Myc*-HisC via *Nco* I and *EcoR* I | This study |
| IscS-H104Q-pBAD/*Myc*-HisC | Amp^r^, the IscS H104Q variant cloned into pBAD/*Myc*-HisC via *Nco* I and *EcoR* I | This study |
| IscS-D180G-pBAD/*Myc*-HisC | Amp^r^, the IscS D180G variant cloned into pBAD/*Myc*-HisC via *Nco* I and *EcoR* I | This study |
| IscS-Q183E-pBAD/*Myc*-HisC | Amp^r^, the IscS Q183E variant cloned into pBAD/*Myc*-HisC via *Nco* I and *EcoR* I | This study |
| IscS-K206A-pBAD/*Myc*-HisC | Amp^r^, the IscS K206A variant cloned into pBAD/*Myc*-HisC via *Nco* I and *EcoR* I | This study |
| IscS-C328S-pBAD/*Myc*-HisC | Amp^r^, the IscS C328S variant cloned into pBAD/*Myc*-HisC via *Nco* I and *EcoR* I | This study |
| IscS-K206A&C328S-pBAD/*Myc*-HisC | Amp^r^, the IscS K206A&C328S variant cloned into pBAD/*Myc*-HisC via *Nco* I and *EcoR* I | This study |
| IscS-R354K-pBAD/*Myc*-HisC | Amp^r^, the IscS R354K variant cloned into pBAD/*Myc*-HisC via *Nco* I and *EcoR* I | This study |
| EH-IscS-pBAD/His SUMO | Amp^r^, the chimeric cysteine desulfurase EH-IscS cloned into pCold I via *Kpn* I | This study |
| HE-IscS-pBAD/His SUMO | Amp^r^, the chimeric cysteine desulfurase HE-IscS cloned into pCold I via *Kpn* I | This study |
| NFS1(55-457)-pBAD/His SUMO | Amp^r^, the human cysteine desulfurase NFS1(55-457) cloned into pBAD/His SUMO via *Nde* I | This study |
| IscS-pCold I | Amp^r^, the IscS cloned into pCold I via *Kpn* I | This study |
| EH-IscS-pCold I | Amp^r^, the chimeric cysteine desulfurase EH-IscS cloned into pCold I via *Kpn* I | This study |
| HE-IscS-pCold I | Amp^r^, the chimeric cysteine desulfurase HE-IscS cloned into pCold I via *Kpn* I | This study |
| EH-IscS-pCold-SUMOa | Amp^r^, the chimeric cysteine desulfurase cloned into pCold-SUMOa via *Nde* I | This study |
| EH-IscS-pCold TF | Amp^r^, the chimeric cysteine desulfurase cloned into pCold TFc via *Kpn* I | This study |
| SufS-pCold I | Amp^r^, the SufS cloned into pCold I via *Kpn* I | This study |
| SufE-pCold I | Amp^r^, the SufE cloned into pCold I via *Kpn* I | This study |
| IscU-pCold I | Amp^r^, the IscU cloned into pCold I via *Kpn* I | Wenzhou Medical University |

**Supplementary Table S2** Primers used for the construction of recombinant plasmids.

| Primer | Sequence (5′→ 3′)^a^ |
| --- | --- |
| IscS-pCold I-F | AGGCATATGGAGCTCATGAAATTACCGATTTATCTCGACTACTCCG |
| IscS-pCold I-R | ATTCGGATCCCTCGAGTTAATGATGAGCCCATTCGATGCTGTTC |
| EH-IscS-pCold-F | AGGCATATGGAGCTCATGAAATTACCGATTTATCTCGACTACTCCG |
| EH-IscS-pCold-R | ATTCGGATCCCTCGAGCTAGTGTTGGGTCCACTTGATGC |
| HE-IscS-pCold I-F | AGGCATATGGAGCTCGTGCTGCGACCTCTCTATATGGATG |
| HE-IscS-pCold I-R | ATTCGGATCCCTCGAGTTAATGATGAGCCCATTCGATGC |
| NFS1(55-457)-pCold-F | AGGCATATGGAGCTCGTGCTGCGACCTCTCTATATGGATG |
| NFS1(55-457)-pCold-R | ATTCGGATCCCTCGAGCTAGTGTTGGGTCCACTTGATGCTC |
| SufS-pCold I-F | AGGCATATGGAGCTCATGATTTTTTCCGTCGACAAAGTGC |
| SufS-pCold I-R | ATTCGGATCCCTCGAGTTATCCCAGCAAACGGTGAATACG |
| SufE-pCold I-F | AGGCATATGGAGCTCATGGCTTTATTGCCGGATAAAG |
| SufE-pCold I-R | AGGCATATGGAGCTCATGGCTTTATTGCCGGATAAAG |
| SUMO-EH-IscS -F | CGAACAGATTGGAGGTATGAAATTACCGATTTATCTCGACTACTCCG |
| SUMO-EH-IscS-R | TCGAGGGTACCGAGCTCCTAGTGTTGGGTCCACTTGATGC |
| SUMO-NFS1(55-457)-F | CGAACAGATTGGAGGTGTGCTGCGACCTCTCTATATGGATG |
| SUMO-NFS1(55-457)-R | TCGAGGGTACCGAGCTCCTAGTGTTGGGTCCACTTGATGCTC |
| IscS-pBAD/Myc-HisC-F | CAGGAGGAATTAACCATGGATGAAATTACCGATTTATCTCGA |
| IscS-pBAD/Myc-HisC-R | CTACGTAAGCTTCGAATTCTTAATGATGAGCCCATTCGA |
| IscS H104Q-pBAD/Myc-HisC-F1 | CCGAACAAAAAGCGGTACTGGATAC |
| IscS H104Q-pBAD/Myc-HisC-R1 | ACTCGTGCACCCAACTGATCTTCAG |
| IscS H104Q-pBAD/Myc-HisC-F2 | GTTGGGTGCACGAGTGGGTTAC |
| IscS H104Q-pBAD/Myc-HisC-R2 | GTACCGCTTTTTGTTCGGTTTTG |
| IscS D180G-pBAD/Myc-HisC-F1 | CTATCACGTTGGTGCAACCCAGAG |
| IscS D180G-pBAD/Myc-HisC-R1 | ACTCGTGCACCCAACTGATCTTCAG |
| IscS D180G-pBAD/Myc-HisC-F2 | GTTGGGTGCACGAGTGGGTTAC |
| IscS D180G-pBAD/Myc-HisC-R2 | GTTGCACCAACGTGATAGATAATGC |
| IscS Q183E-pBAD/Myc-HisC-F1 | ACCGAGAGCGTGGGTAAACTGC |
| IscS Q183E-pBAD/Myc-HisC-R1 | ACTCGTGCACCCAACTGATCTTCAG |
| IscS Q183E-pBAD/Myc-HisC-F2 | GTTGGGTGCACGAGTGGGTTAC |
| IscS Q183E-pBAD/Myc-HisC-R2 | TACCCACGCTCTCGGTTGCA |
| IscS K206A-pBAD/Myc-HisC-F1 | GTCACGCAATCTATGGCCCGAAAGG |
| IscS K206A-pBAD/Myc-HisC-R1 | ACTCGTGCACCCAACTGATCTTCAG |
| IscS K206A-pBAD/Myc-HisC-F2 | GTTGGGTGCACGAGTGGGTTAC |
| IscS K206A-pBAD/Myc-HisC-R2 | GCCATAGATTGCGTGACCGG |
| IscS C328S-pBAD/Myc-HisC-F1 | CCTCTACGTCAGCAAGCCTCGAAC |
| IscS C328S-pBAD/Myc-HisC-R1 | ACTCGTGCACCCAACTGATCTTCAG |
| IscS C328S-pBAD/Myc-HisC-F2 | GTTGGGTGCACGAGTGGGTTAC |
| IscS C328S-pBAD/Myc-HisC-R2 | GCTTGCTGACGTAGAGGCGG |
| IscS R354K-pBAD/Myc-HisC-F1 | ATCAAGTTCTCTTTAGGTCGTTTTACTACTG |
| IscS R354K-pBAD/Myc-HisC-R1 | TGACGCTCAGTGGAACGAAAAC |
| IscS R354K-pBAD/Myc-HisC-F2 | GTTCCACTGAGCGTCAGACCC |
| IscS R354K-pBAD/Myc-HisC-R2 | CGACCTAAAGAGAACTTGATAGAGCTATG |

# Supplementary Figures


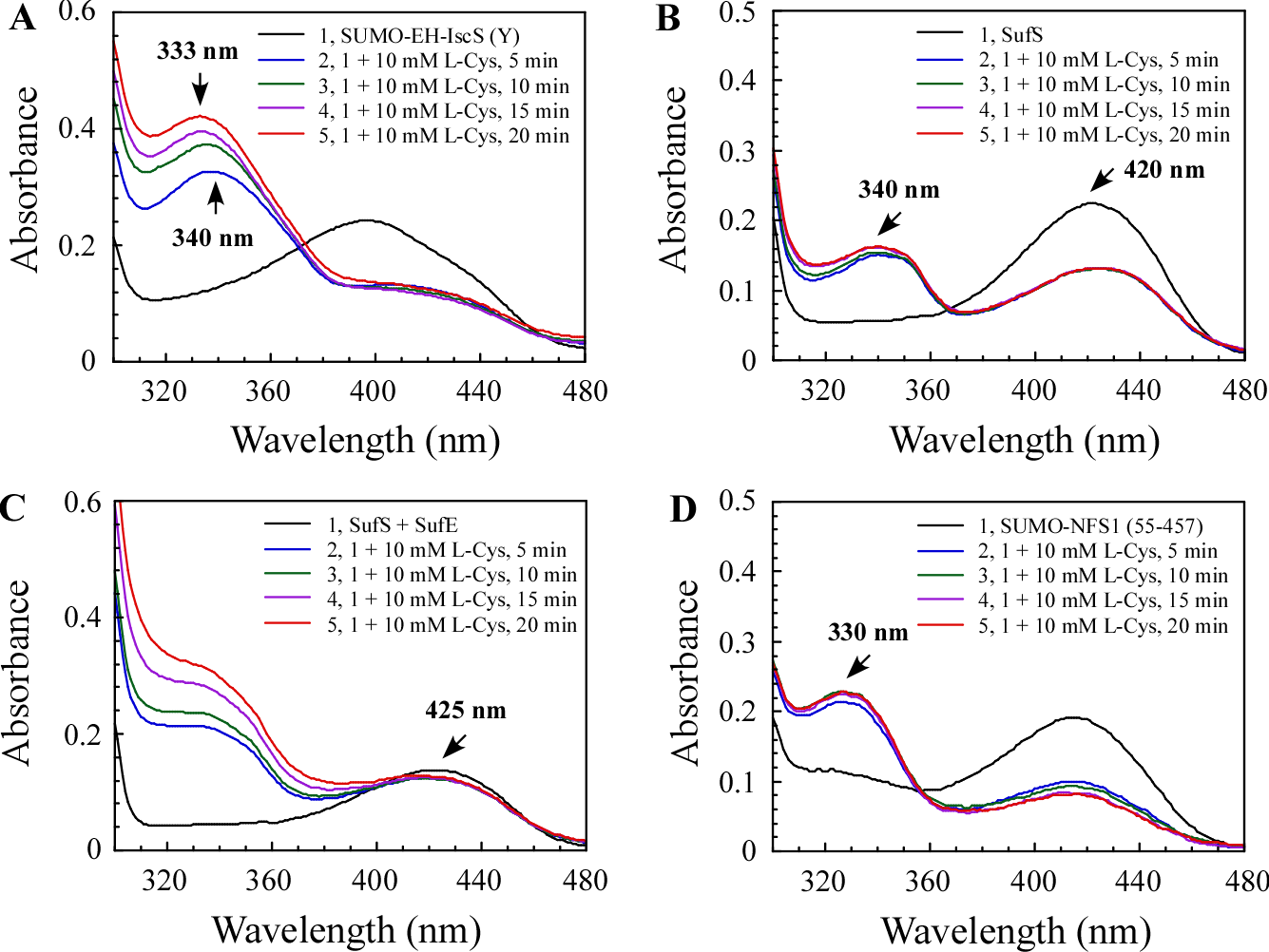


**Supplementary Figure S1** UV-visible spectral changes of recombinant CDs upon the addition of 10 mM L-cysteine. **(A-D)** Purified SUMO-EH-IscS (A), SufS (B), SufS + SufE (C), and SUMO-NFS1 (55-457) (D) were incubated with 10 mM of L-cysteine at room temperature. The UV-visible absorption spectrum was recorded every 5 min for 20 min. All protein concentrations were calibrated to about 40 μM with buffer A, except SufE (160 μM). The data are representative of three independent experiments.


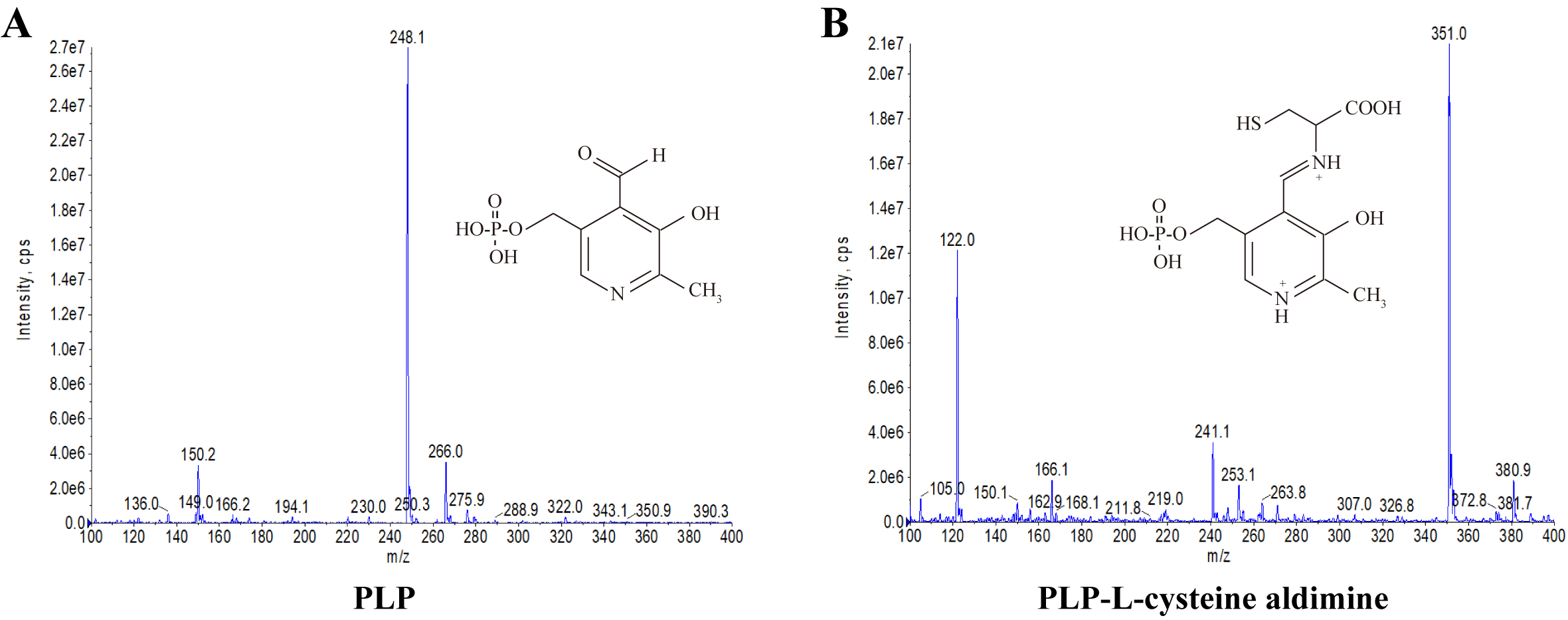


**Supplementary Figure S2** UPLC-MS spectra of PLP and its derivatives. **(A)** Full-scan parent ion spectra of PLP standard (E); **(B)** PLP-L-cysteine aldimine was derived from the reaction product of PLP and L-cysteine. The inserts in (A) and (B) are the chemical structures of PLP and PLP-L-cysteine aldimine. The masses of 248.1 Da and 351 Da correspond to PLP (A) and PLP-L-cysteine aldimine (B), respectively.


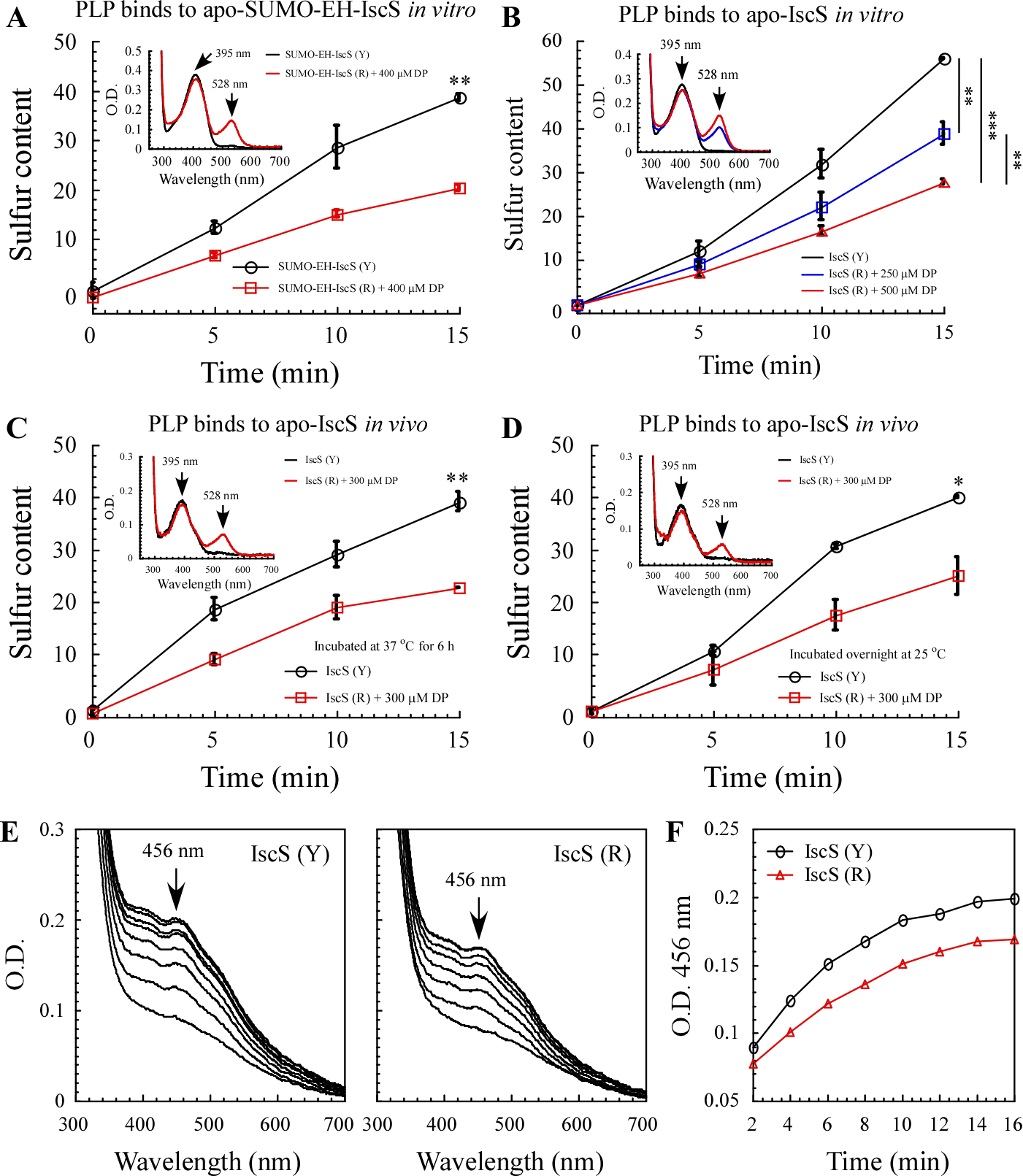


**Supplementary Figure S3** Effects of red intermediate accumulation on recombinant CD activity and iron-sulphur cluster assembly within IscU *in vitro*. The amplitudes of the PLP absorption peak at 395 nm for the equimolar concentrations of the red and yellow CDs are in agreement, and the activity of the two CDs is the same. Therefore, if there is a difference in activity between the yellow and red CDs, the difference is most likely due to the accumulation of red intermediates (528 nm) in CDs. Recombinant CDs were expressed in *E. coli* BL21(DE3) cells grown in LB media with or without different concentrations of 2,2’-dipyridyl (DP). When the cell density reached OD_600_ of 0.6, recombinant protein was induced with 0.02% arabinose at 25°C for 24 h (A-B)/overnight (D) or at 37 °C for 6 h (C). **(A-D)** Comparison of L-cysteine desulphurisation activity of red and yellow CDs after the overexpressed CDs were incubated with PLP *in* *vitro* or *in vivo*. (A-B) Studies have shown that many of the overexpressed IscS is apoproteins (Tan et al. 2014). Moreover, under cold stress conditions, the proportion of apoproteins will be higher (Ren et al. 2021). Therefore, before a comparative analysis of yellow and red IscS or yellow and red SUMO-EH-IscS activity differences, they were first incubated with excess PLP *in vitro* to reach PLP saturation in each protein. The purified IscS (Y) and IscS (R) were incubated with 1 mM of PLP on ice for 30 min and the PLP cofactor bound by the recombinant CDs reached saturation and were re-purified by passing through a HiTrap desalting column. Therefore, the desalted yellow and red CDs have the same amplitudes of the absorption peak at 395 nm. (C-D) The activity of yellow and red IscS was analysed under the condition where the overexpressed apo-IscS-bound PLP was saturated by adding exogenous PLP (80 mg/L) to the LB medium during inoculation; **(E-F)** Red IscS modulated the iron-sulphur cluster assembly within IscU *in vitro*. Purified IscS (Y) and IscS (R) proteins were obtained from (C). The absorption peak at 456 nm was used to indicate the amount of the [2Fe-2S] clusters assembled in IscU. The data are presented as the mean ± SD of at least three independent experiments. * Indicates *p* < 0.05, ** indicates *p* < 0.01, and *** indicates *p* < 0.001.
